# Retinal layer structure on optical coherence tomography in post-traumatic stress disorder

**DOI:** 10.1371/journal.pone.0360471

**Published:** 2026-10-27

**Authors:** Sejal D. Patel, Naveen Karthik, Grant A. Justin, Alice Haystead, Stephanie J. Chiu, Sandra S. Stinnett, Kim G. Johnson, Dilraj S. Grewal, Sharon Fekrat

**Affiliations:** 1 Department of Ophthalmology, iMIND Study Group, Duke University School of Medicine, Durham, NC, United States of America; 2 Walter Reed National Military Medical Center, Bethesda, MD, United States of America; 3 Department of Neurology, Duke University School of Medicine, Durham, NC, United States of America; Astellas, UNITED STATES OF AMERICA

## Abstract

**Purpose:**

To assess individual retinal layer thickness and volume changes on optical coherence tomography (OCT) in individuals with post-traumatic stress disorder (PTSD) compared to controls.

**Methods:**

Adults ≥18 years with a PTSD diagnosis were identified. Age- and sex-matched controls (±5 years) without PTSD were recruited. Exclusion criteria included any cognitive disorder, brain injury, glaucoma, diabetes, optic nerve or vitreoretinal disease, uncontrolled hypertension, refractive error >±6 diopters, or best corrected visual acuity <20/40. Non-mydriatic OCT macular cube 512x128 images were obtained using Zeiss Cirrus HD-5000 AngioPlex. The Duke Reading Center DRCVisualizer software was used to segment the ganglion cell/inner plexiform (GC-IPL), inner nuclear (INL), outer plexiform (OPL), outer nuclear (ONL), photoreceptor, and retinal pigment epithelium (RPE) layers. Mean retinal thickness and volume were calculated for all layers within the central 1-mm subfield and 3- and 6-mm ETDRS rings, further divided into nasal, superior, temporal, and inferior quadrants.

**Results:**

81 eyes of 42 PTSD patients and 81 eyes of 42 controls were analyzed. Average ages were 48 ± 15 years for PTSD and 51 ± 19 years for controls. Groups were 36% male. PTSD eyes showed significantly decreased photoreceptor thickness and volume and increased RPE thickness/volume across all ETDRS rings and quadrants (all p < 0.05). OPL thicknesses were decreased in PTSD eyes versus controls for temporal 3-mm (25.29 ± 1.36 µm vs. 26.64 ± 3.80µm) and temporal 6-mm (23.47 ± 0.88µm vs. 24.00 ± 1.54µm) rings (p < 0.05). OPL inferior 6-mm thickness was decreased in PTSD eyes (23.98 ± 1.58 µm vs. 24.78 ± 2.34 µm) versus controls (p < 0.05). The ONL nasal 6-mm thickness was decreased in PTSD versus controls (55.96 ± 7.90 µm vs. 56.74 ± 7.47µm) (p < 0.05). After adjusting for multiple testing, RPE differences remained significant across all evaluated regions, while photoreceptor differences remained significant within the central 1-mm and 3-mm ETDRS regions.

**Conclusions:**

PTSD eyes demonstrated decreased photoreceptor layer and increased RPE thickness and volume.

## Introduction

Post-traumatic stress disorder (PTSD) is a chronic condition associated with long-term neurobiological changes, including dysregulation of the hypothalamic-pituitary-adrenal axis and alterations in neurovascular function [1]. PTSD is represented by emotional memories, avoidant behavior, mood fluctuation, and heightened arousal [2]. Studies have revealed diverse rates of PTSD across different populations and geographic regions due to varying levels of access to care, education about PTSD, and self-biased assessments. The United States (US) Department of Veteran Affairs estimates the lifetime risk of PTSD at 6% in the general US population [3]. However, this rate varies significantly depending on the context and exposure to trauma. For instance, in conflict-affected populations (such as war-ridden communities), the pooled prevalence of PTSD was higher, up to about 30% [4]. There is a substantial economic burden associated with PTSD. One analysis estimated the cost of PTSD in the US to be $232.2 billion in 2018, with a projected upward trend, primarily due to the spike in PTSD cases due to the stressors associated with the COVID-19 pandemic [5].

PTSD is associated with differences in brain structure and function. Neuroimaging studies have consistently shown alterations in key brain regions implicated in the stress response [6,7]. Meta-analyses have revealed individuals with PTSD exhibit a 6.9% smaller hippocampal volume compared to trauma-exposed controls [8]. Additionally, studies have found on average, smaller volumes of the anterior cingulate in PTSD. This structure plays a crucial function in modulating retinal input via the optic nerve and lateral geniculate nucleus. Thus, changes in the anterior cingulate due to PTSD may be associated with alterations in retinal structure [9,10]. Furthermore, the hypothalamus and thalamus in PTSD have been found to have decreased volume [11,12]. The hypothalamus originates from the diencephalon during fetal brain development, which is also the structure that gives rise to the retina [11,12]. Given the commonality in embryologic origin of the hypothalamus and retina, a key area of study is understanding how PTSD is correlated to changes within the retina [13,14]. Prior research has highlighted retinal layer atrophy associated with several neuropsychiatric conditions, including schizophrenia, bipolar disorder, and major depressive disorder [15]. The reported changes include decreased macular volume and retinal nerve fiber layer thickness observed on optical coherence tomography (OCT) [15]. Prior studies have found thinning of the ganglion cell-inner plexiform layer and outer plexiform layer, and thickening of the RPE layer, in neurodegenerative diseases such as Alzheimer’s Disease, Parkinson’s Disease, glaucoma, and age-related macular degeneration [16,17]. There have also been observations of increased RPE layer thickness in individuals with moderate traumatic brain injury (TBI) as compared to more severe cases of TBI [18].

OCT has shown promise as a non-invasive approach in quantifying retinal structures, including retinal layer thickness and volume, to detecting early signs of neurodegeneration in various conditions, raising the possibility of its utility as a biomarker and severity measure in PTSD research [19,20]. This study aims to investigate retinal structural findings in eyes of patients with PTSD using OCT imaging, specifically focusing on alterations in the thickness and volume of the individual retinal layers. The information gathered from retinal layer thickness and volume measurements may reveal important imaging biomarkers that can help identify and potentially monitor PTSD.

## Methods

This study of PTSD subjects was approved by the Institutional Review Board of the Duke University Health System and was registered with ClinicalTrials.gov (NCT03233646). All study activities adhered to the tenets of the Declaration of Helsinki and to regulations outlined in the Health Insurance Portability and Accountability Act. Written consent was obtained from participants prior to enrollment. The enrollment period was from 8/21/2023 to 03/01/2026.

### Recruitment and inclusion criteria

Adults aged 18 years and older diagnosed with PTSD by a psychiatrist or primary care physician between January 1, 2018 and December 31, 2021 within the Duke University Health System were identified through the Duke Enterprise Data Unified Content Explorer (DEDUCE). Participants were recruited via email or phone. Inclusion criteria mandated that participants have a confirmed PTSD diagnosis using ICD-10 diagnosis codes (F43.1, F43.10, F43.11, F43.12). Age- and sex-matched controls without PTSD were enrolled for comparative analysis. Controls were matched within ± 5 years of age of the PTSD cohort participants.

### Exclusion criteria

Participants were ineligible for the study if they had any cognitive impairment, including early-stage memory loss, neurodegenerative conditions affecting cognition, or any dementia. The study also excluded individuals with a history of direct head trauma, substance-related damage, or oxygen deprivation. Those with diabetes mellitus or uncontrolled hypertension, defined as hypertension persisting after medical treatment, were excluded. Ophthalmic conditions excluded were glaucoma, glaucoma suspect, optic nerve pathology, and any vitreoretinal disorder. Individuals with corrected visual acuity worse than 20/40 or with significant refractive error exceeding +6 or −6 diopters were excluded. The study also excluded anyone who had undergone any intraocular eye surgery, except for uncomplicated cataract surgery. In cases where only one eye met the study requirements, data from that eye alone was used in the analysis. OCT images were excluded if they exhibited poor scan quality (signal strength <7/10) due to low resolution, poor saturation, reflections, media opacity, or motion artifacts resulting from inadequate patient cooperation [20]. Multiple images of the same eye from patients gathered at different timepoints were excluded from the study.

### Data collection and analysis

Participants underwent undilated OCT imaging and assessment by trained Eye Multimodal Imaging in Neurodegenerative Disease (iMIND) study staff [21,22]. This included best corrected visual acuity (BCVA) measurements and Mini-Mental State Examination (MMSE) for each participant to confirm cognitive status. All participants identified as cognitively normal and attained MMSE scores in a cognitively normal range [23]. OCT imaging of the macula (macular cube 512x128) centered at the fovea was performed using a Zeiss Cirrus HD-5000 Spectral-Domain OCT AngioPlex (version 11.0.0.29946, Carl Zeiss Meditec, Dublin, CA). The Duke Reading Center DRCVisualizer software (version number 2.1.1, Duke Reading Center, Durham, NC), was then used to segment the individual retinal layers on the OCT macular images, including the ganglion cell and inner plexiform layer (GC-IPL), inner nuclear layer (INL), outer plexiform layer (OPL), outer nuclear layer (ONL), photoreceptor layer, defined by the inner segment ellipsoid zone (EZ) and outer segment, and retinal pigment epithelium (RPE) layer. The software uses a graph-based image segmentation algorithm that assigns weights to image nodes to find paths of least resistance. These paths correspond to the various retinal layer boundaries [24]. To ensure accuracy, two trained readers (S.D.P. and N.K.) manually reviewed each scan and corrected segmentations as needed, with input from experienced adjudicators (D.S.G. and S.J.C.). Mean retinal layer thickness (in micrometers) and volume (in cubic millimeters) were calculated for all specified retinal layers within the central 1-mm subfield of the foveal center point and 3- and 6-mm Early Treatment Diabetic Retinopathy Study (ETDRS) rings. The 3- and 6-mm rings were further subdivided into nasal, superior, temporal, and inferior quadrants (Fig 1). Within the ETDRS rings, the nasal region is defined as being nasal to the fovea and temporal to the optic disc.

**Fig 2:**
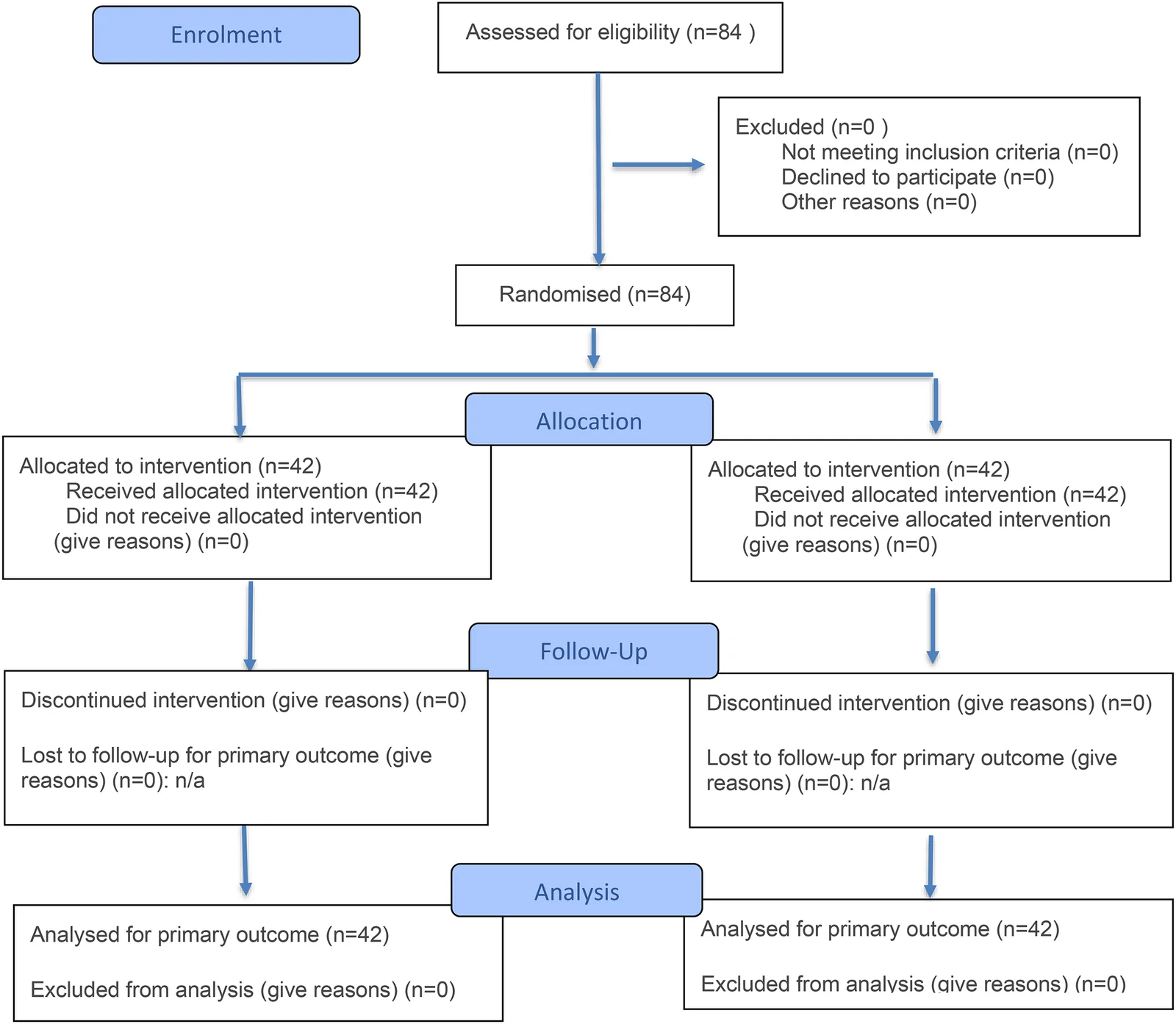
CONSORT 2025 flow diagram. Flow diagram of the progress through the phases of a randomised trial of two groups (that is, enrolment, intervention allocation, follow-up, and data analysis).

**Fig 1:**
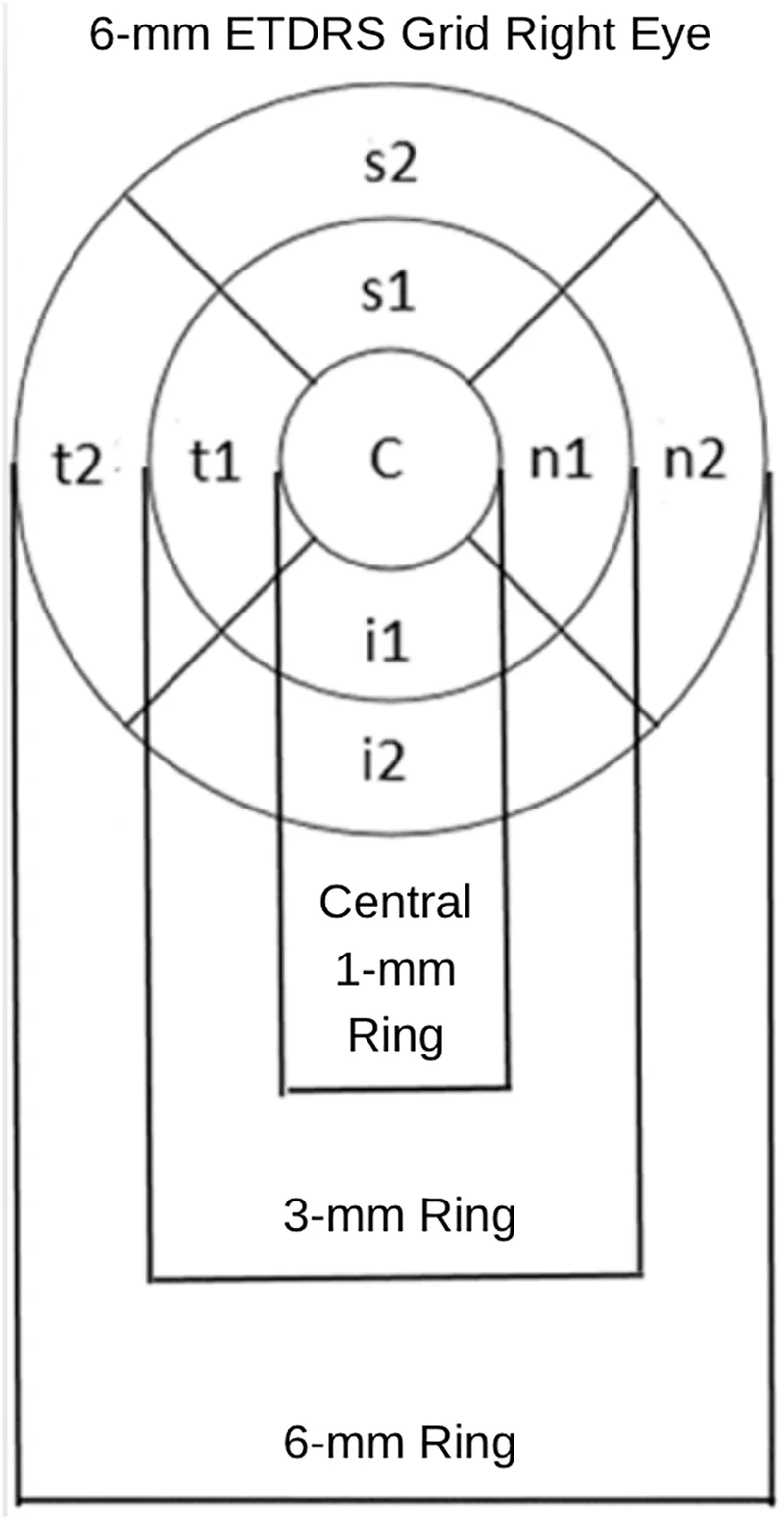
An example diagram of ETDRS 1-mm, 3-mm, and 6-mm rings of the right eye.

Data analysis was performed using SAS/STAT software Version 9.4 of the SAS System for Windows (SAS Institute Inc., Cary, NC). Comparisons between groups based on patient-level demographics were conducted using the Wilcoxon rank sum test for continuous variables and Fisher’s exact test for categorical variables. Statistical analysis was conducted using generalized estimating equations (GEE) analysis with a significance level set at α = 0.05. GEE models with an exchangeable correlation structure were specifically selected to account for inter-eye correlation when both eyes from a participant met eligibility criteria. Cohen’s d estimates were calculated to examine the effect sizes of each retinal finding. Bonferroni correction was applied separately within each retinal layer to account for multiple comparisons across retinal measurements, with 19 thickness and volume comparisons comprising each family of tests. This yielded a Bonferroni-adjusted significance threshold of p < 0.0026 (0.05/19). Bonferroni-adjusted P-values were calculated by multiplying each unadjusted p-value by 19, with values exceeding 1.0 reported as 1.0. A two-sided unadjusted p-value < 0.05 was considered statistically significant before correction for multiple comparisons. Given the modest sample size and individual matching of controls to PTSD participants by age and sex, propensity score matching was not additionally performed.

## Results

A total of 162 eyes of 84 total participants were included, including 81 eyes from 42 patients with PTSD and 81 from 42 age- and sex-matched controls (Fig 2). The average age of the controls was 51.1 ± 19.1 years, and of the PTSD patients was 47.9 ± 15.0 years. 35.7% of PTSD patients and controls were male. The average MMSE score for PTSD patients was 29.2 ± 1.4 (out of 30), and for controls was 29.5 ± 0.9, indicating each group was within the cognitively normal range (Table 1).

**Table 1:** Demographical data for PTSD cohort participants and age-/sex-matched controls.

Demographics				
Variable	Statistic	PTSD	Control	P-value*
Age (years)	N	42	42	
	Mean (SD)	47.9 (15.0)	51.1 (19.1)	
	Min, Median, Max	21.03, 50.14, 79.27	19.24, 57.12, 83.83	0.187
Mini Mental State Exam score	N	42	42	
	Mean (SD)	29.2 (1.4)	29.5 (0.9)	
	Min, Median, Max	23.0, 30.0, 30.0	26.0, 30.0, 30.0	0.203
Years of education	N	42	42	
	Mean (SD)	17.05 (2.34)	16.7 (2.5)	
	Min, Median, Max	12.0, 16.0, 22.0	12.0, 16.0, 22.0	0.286
Race	N (%)			0.367
Asian		1 (2.38)	5 (11.9)	
Black		5 (11.9)	2 (4.76)	
White Other/Not Reported		35 (83.3) 1 (2.38)	33 (78.57) 2 (4.76)	
Female Sex	N (%)	27 (64.3)	27 (64.3)	1.000

*P-values for continuous variables based on Wilcoxon rank sum test. *P*-value for categorical variables based on Fisher’s exact test. PTSD = Post-Traumatic Stress Disorder.

PTSD eyes demonstrated consistently greater thickness and volume compared to control eyes across all measured regions of the RPE layer, including the center point and nasal, superior, temporal, and inferior 3-mm and 6-mm perifoveal rings and quadrants (all p < 0.005) (S1 Table). PTSD eyes had a significantly increased mean center 1-mm thickness of the RPE layer (29.18 ± 3.87 µm) compared to controls (24.10 ± 6.09 µm) (p < 0.001) (S1 Table). PTSD eyes also demonstrated increased volume (0.023 ± 0.003 mm^3^) compared to control eyes (0.019 ± 0.005 mm^3^) in the center 1-mm ring (p < 0.001) (S1 Table). All RPE comparisons remained statistically significant following Bonferroni correction (all Bonferroni-adjusted p < 0.019) (S2 Table).

The photoreceptor layer exhibited decreased mean thickness and volume measurements in PTSD eyes compared to control eyes across all measured retinal regions (all p < 0.05), although only the central 1-mm and 3-mm findings remained statistically significant following Bonferroni correction (all Bonferroni-adjusted p ≤ 0.019) (S1 Table and S2 Table). The center 1-mm thickness of the photoreceptor layer was significantly decreased in PTSD eyes (57.72 ± 5.26 µm) compared to control eyes (62.16 ± 7.07 µm) (p = 0.001) (S1 Table). Similarly, the mean center 1-mm volume was significantly decreased in PTSD eyes (0.045 ± 0.004 mm^3^) compared to controls (0.049 ± 0.006 mm^3^) (p = 0.001) (S1 Table).

The OPL demonstrated reductions in thickness and volume in the temporal 3- and 6-mm and inferior 6-mm regions that met the unadjusted significance threshold. In the temporal 3-mm ring, PTSD eyes showed significantly reduced thickness (25.29 ± 1.36 µm) compared to control eyes (26.64 ± 3.80 µm) (p = 0.013) (S1 Table). Volume of the temporal 3-mm ring in PTSD eyes (0.040 ± 0.002 mm^3^) was also reduced compared to controls (0.042 ± 0.006 mm^3^) (p = 0.013) (S1 Table). Nasal, superior, and inferior 3-mm thickness and volume measurements were not statistically different (all p > 0.10) (S1 Table). PTSD eyes again showed significantly reduced thickness compared to control eyes in the temporal 6-mm perifoveal ring (23.47 ± 0.88 µm vs 23.99 ± 1.54 µm) (p = 0.026) and the inferior 6-mm ring (23.98 ± 1.58 vs 24.78 ± 2.34 µm) (S1 Table). Volume in the temporal 6-mm perifoveal ring was reduced in PTSD eyes compared to control eyes (0.12 ± 0.005 mm^3^ vs 0.13 ± 0.008 mm^3^) (p = 0.026). Similarly, volume was reduced in PTSD eyes (0.127 ± 0.008 mm^3^) versus control eyes (0.131 ± 0.012 mm^3^) in the inferior 6-mm ring (p = 0.035) (S1 Table). Nasal and superior 6-mm regions did not show significant differences in thickness or volume (all p > 0.30) (S1 Table). The mean center 1-mm thickness and volume were not significantly different between PTSD eyes compared to controls (p > 0.08). None of these OPL differences remained statistically significant following Bonferroni correction (adjusted p = 0.247–0.665) (S2 Table).

No significant difference was noted in ONL center mean 1-mm thickness in PTSD eyes (91.24 ± 10.50 µm) compared to controls (92.44 ± 10.46 µm) (p = 0.576) (S1 Table). The ONL mean center 1-mm volume was 0.072 ± 0.008 mm^3^ while the control group was 0.073 ± 0.008 mm^3^ (p = 0.576) (S1 Table). However, the ONL nasal 6-mm thickness was significantly reduced in PTSD eyes (55.96 ± 7.90 µm) compared to controls (56.74 ± 7.47 µm) (p = 0.039), along with nasal 6-mm volume in PTSD eyes (0.297 ± 0.04 mm^3^) versus controls (0.301 ± 0.04 mm^3^) (p = 0.039) (S1 Table). Similar to the OPL, the reduction in ONL nasal 6-mm thickness and volume met the unadjusted significance threshold but did not remain significant following Bonferroni correction (both adjusted p = 0.741) (S1 and S2 Table). All other measured regions of the ONL did not exhibit significant differences.

Other retinal layers did not demonstrate statistically significant differences between PTSD eyes and controls. The GC-IPL showed no significant variation in mean central 1-mm thickness in PTSD eyes (35.50 ± 10.86 µm) compared to controls (39.37 ± 13.20 µm) (p = 0.076) (S1 Table). The GC-IPL mean center 1-mm volume was 0.028 ± 0.009 mm^3^ while the control group was 0.031 ± 0.010 mm^3^ (p = 0.076) (S1 Table). In the INL, there were no significant differences in mean central 1-mm thickness between PTSD eyes (20.83 ± 5.45 µm) and controls (21.48 ± 4.56µm) (p = 0.209) (S1 Table). The INL mean center 1-mm volumes were the same across controls and PTSD eyes (0.02 ± 0.004 mm^3^) (p = 0.209) (S1 Table).

There were no statistically significant differences in mean total retinal thickness or volume between PTSD and control eyes at the center point, central 1-mm region, or across any nasal, superior, temporal, or inferior 3-mm and 6-mm perifoveal regions (all p > 0.18) (S1 Table). Mean center 1-mm retinal thickness was 273.83 ± 21.26 µm in PTSD eyes compared to 280.59 ± 23.39 µm in controls (p = 0.220). Similarly, mean center 1-mm retinal volume did not differ significantly between PTSD eyes (0.215 ± 0.017 mm^3^) and controls (0.220 ± 0.018 mm^3^) (p = 0.220) (S1 Table).

Standardized mean differences demonstrated large effects for RPE thickness (Cohen’s d = 0.76–1.00), moderate to large effects for photoreceptor thickness (|d| = 0.45–0.78), smaller effects for OPL thickness (|d| = 0.40–0.47), and a very small effect for ONL thickness (d = −0.10).

## Discussion

Our study of retinal layer thickness and volume measurements in eyes of patients with PTSD demonstrated photoreceptor thinning and RPE thickening, with the strongest differences remaining significant after correction for multiple comparisons. Differences were also observed within the OPL and ONL that met the unadjusted significance threshold but did not remain significant following Bonferroni correction (S2 Table).

Some studies hypothesize a weak relationship between dopaminergic neurons (which are impaired in PTSD) and photoreceptor cells, characterized by loss of typical dopaminergic control over the communication between photoreceptor cells [25–27]. Previous studies in PTSD patients suggest abnormal dopaminergic neuron release in response to stressors that are correlated with PTSD [27]. Previous literature has reported selective layer thinning of the photoreceptor layer in the eyes of individuals with Parkinson’s disease, where dopaminergic neurons are impaired [27]. One study compared individuals with Parkinson’s disease to controls without Parkinson’s and found significant combined outer nuclear and photoreceptor layer thinning in Parkinson’s disease patients compared to controls. Given that dopaminergic dysfunction has been implicated in both PTSD and Parkinson’s disease, we postulate a potential relationship between photoreceptor layer thinning and dopaminergic impairment seen in both disease populations, which would benefit from further investigation [27,28].

The photoreceptor findings were among the most robust structural differences observed in this study. Following Bonferroni correction, decreased photoreceptor thickness and volume remained statistically significant within the central 1-mm and all four 3-mm ETDRS regions, whereas the center-point and 6-mm differences did not remain significant (S2 Table). The observed thinning of the photoreceptor layer in PTSD is possibly explained by several factors associated with trauma-related conditions, including neuroendocrine dysregulation and oxidative stress [29–34]. PTSD is associated with dysregulation of the HPA axis, which may contribute to the neuroendocrine and neurovascular alterations [29]. Chronic cortisol release can increase oxidative stress, which is particularly damaging to photoreceptors due to their high metabolic rate and frequent light exposure [30]. The chronic low-grade inflammation associated with PTSD may extend to the retina, potentially contributing to photoreceptor degeneration over time [29]. Photoreceptor cells are heavily dependent on a balance between blood supply and oxygen consumption. PTSD has been associated with autonomic dysfunction, leading to fluctuation of retinal blood flow, which may contribute to poor photoreceptor health and decreased survival [31,32]. Because the ONL is primarily composed of nuclei of cone and rod photoreceptors, photoreceptor cell death in PTSD may be related to the ONL thinning observed in this population; however, the nasal 6-mm ONL difference observed before correction did not remain significant after Bonferroni correction and should be interpreted cautiously [35].

RPE alterations represented the most consistent structural finding in this study, with increased RPE thickness and volume remaining statistically significant across all evaluated regions after Bonferroni correction. RPE thickening may arise from several mechanisms including individual RPE cellular hypertrophy, hyperplasia, or the accumulation of cellular debris [33]. Similar mechanisms to photoreceptor thinning may also contribute to RPE layer thickening including cortisol-induced chronic oxidative stress. Chronic oxidative stress associated with PTSD could contribute to mitochondrial dysfunction in RPE cells, potentially promoting cellular swelling and increased RPE thickness [34]. Additionally, the inflammatory state of PTSD may trigger RPE cell hypertrophy or hyperplasia as a protective mechanism to combat inflammation [36,37]. The RPE layer thickening might also be a compensatory response to photoreceptor layer thinning, as the RPE layer attempts to maintain support for the remaining photoreceptors by producing more cytokines and growth factors (including pigment epithelium-derived growth factor, vascular endothelial growth factor, and/or lens epithelium-derived growth factor) to maintain survival of photoreceptors [38]. Furthermore, PTSD-related stress may affect the phagocytic function of RPE cells, leading to an accumulation of cellular debris and contributing to RPE layer thickening [39].

Similar findings have been documented in AMD, where OCT studies reveal RPE thickening alongside photoreceptor thinning [40]. In AMD, thickening is attributed not only to hypertrophy and debris accumulation but also to the buildup of extracellular material such as drusen beneath the RPE and within subretinal drusenoid deposits above the RPE layer [41–43]. Subretinal drusenoid deposits increase oxidative stress and inflammation, progressively impairing photoreceptor and RPE function in AMD [43]. These findings suggest possible overlapping mechanisms between PTSD and AMD, involving oxidative damage, inflammation, extracellular material accumulation, and decreased macular perfusion that could drive pathological RPE and photoreceptor changes [44].

The findings of OPL thinning in the temporal and inferior rings met the unadjusted significance threshold but did not remain statistically significant following Bonferroni correction. Given the OPL contains the synapses between photoreceptor cells and bipolar cells, these findings may be explained by similar mechanisms as prior layers given the functional continuity between the OPL and the photoreceptor layer [45]. Repeated mechanisms of chronic microvascular instability and low-grade inflammation that degenerate photoreceptors may extend their effects to the OPL through remodeling and retraction of synapses in the photoreceptor layer [46,47]. However, given that these differences did not remain significant after correction for multiple comparisons, further studies are needed to determine whether OPL alterations are associated with PTSD.

The multiple-comparison analysis was consistent with the observed effect-size pattern. RPE alterations demonstrated predominantly large effects (Cohen’s d = 0.76–1.00) and remained significant across all evaluated regions following Bonferroni correction. Photoreceptor alterations demonstrated moderate to large effects (|d| = 0.45–0.78), with differences in the central 1-mm and 3-mm regions remaining significant after correction. In contrast, the OPL differences that met the unadjusted significance threshold demonstrated smaller effects and did not remain significant after correction, while the ONL nasal 6-mm difference demonstrated a very small effect and similarly did not remain significant. Given the variability of effect sizes seen in retinal layers, further validation is needed before clinical applications. Published studies using spectral-domain OCT have demonstrated high reproducibility of retinal thickness measurements, although test-retest variability varies by retinal layer, region, and segmentation method [48]. Terry et al., for example, reported inter-session coefficients of repeatability of 3.43 µm for foveal total retinal thickness using Zeiss OCT with an automated segmentation algorithm and 4.63 µm using the device’s commercial segmentation software [48]. Because comparable repeatability thresholds have not been established for the manually corrected individual retinal layer measurements used in the present study, we cannot definitively determine whether each observed difference exceeds expected measurement variability. Accordingly, the standardized effect sizes characterize the magnitude of between-group differences but should not be interpreted as establishing clinical significance. Longitudinal studies relating these structural changes to visual function, PTSD severity, and disease progression will be necessary to determine their clinical relevance.

Our study has limitations that should be considered when interpreting the results. First, the cross-sectional nature of this study limits our ability to establish causality or track the progression of retinal findings over time. Longitudinal data were not available for this analysis, limiting potential insights into the temporal relationship between PTSD onset and retinal alterations and changes in retinal structure with PTSD progression or treatment. Due to the lack of data on the precise origin, timing of development, and severity of PTSD in participants, there are confounders in our study. PTSD severity scores were not available because participants were identified through ICD-10 diagnoses within the health system. The specific character of PTSD, including the intensity of symptoms experienced, may influence the downstream neurological and ophthalmic effects [49]. This represents a potential confounder because retinal structural measurements have been associated with psychiatric disease severity in other populations. For example, in patients with major depressive disorder, greater depressive symptom severity was associated with reduced outer nuclear layer thickness and reduced ganglion cell and inner plexiform layer thickness and volume [50]. Additionally, medication data were not systematically collected and therefore could not be incorporated into the analyses. Psychiatric medications commonly used in PTSD act on serotonergic, noradrenergic, and adrenergic pathways that may influence vascular physiology [51]. Further areas of investigation include exploring sub-analyses of the effect of trauma type such as combat-related, childhood trauma, or civilian accidents, or correlations between symptom severity and chronicity and the extent of retinal alterations. Moreover, our study population may not be fully representative of the broader PTSD population. The sample primarily consisted of White adults with access to healthcare, which could introduce selection bias and limit the generalizability of the findings to more diverse populations or those in limited healthcare resource settings. Our study did not account for potential confounding factors such as non-PTSD-related medication use, lifestyle factors such as smoking, alcohol consumption, drug use, or comorbid medical conditions that may affect retinal structure. Finally, each OCT scan required manual correction after analysis by the semi-automatic retinal segmentation software, which may have presented subtle variations within the thickness and volume results. For the primary cohort of 51 participants (25 individuals with PTSD and 26 cognitively normal controls), manual segmentation was not masked by participant cohort group; for the additional 33 participants added after 2023 (17 individuals with PTSD and 16 cognitively normal controls), manual segmentation was masked for graders (SDP). Intraobserver and interobserver reproducibility were not formally assessed, and reader-dependent variability in retinal layer segmentation therefore cannot be excluded. However, both readers independently followed the same predefined segmentation protocol and standardized criteria for identifying retinal layer boundaries to promote consistency across measurements.

This study demonstrates significant retinal structural changes in individuals with PTSD, with the strongest findings involving thinning of the photoreceptor layer and thickening of the RPE. RPE differences remained significant across all evaluated regions following correction for multiple comparisons, while photoreceptor differences remained significant within the central 1-mm and 3-mm ETDRS regions. While other retinal layers did not show widespread significant differences in thickness and volume, the specific changes in the photoreceptor and RPE layers highlight the potential impact of PTSD on retinal structure. These findings add to the literature on the potential effects of PTSD on the retina and may contribute to our understanding of the systemic effects of PTSD. Furthermore, these findings highlight retinal imaging as a potential non-invasive method for identifying biomarkers in PTSD patients. Further research can investigate the mechanisms underlying these retinal changes and explore the clinical significance of retinal architecture in PTSD diagnosis, monitoring, and treatment.

## Supporting information

S1 Table Appendix A. The following tables list results of average thickness (in micrometers, µm) and volume (in cubic millimeters, mm^3^) measurement comparisons of retinal layers between post-traumatic stress disorder (PTSD) eyes and age-/sex-matched controls. Standard deviation (SD) is noted for parameters along with minimum (min), median, and maximum (max) values. Statistically significant p-values (< 0.05) are bolded.
(DOCX)

S2 Table Appendix B. The following table summarizes retinal layer thickness and volume measurements that differed significantly between post-traumatic stress disorder (PTSD) eyes and age-/sex-matched controls at the unadjusted significance level (p < 0.05). Absolute and percentage differences between group means are provided to characterize the magnitude of observed differences. An asterisk (*) denotes findings that remained statistically significant after Bonferroni correction (adjusted significance threshold, p < 0.0026).
(DOCX)

S3 TREND Checklist. TREND Statement checklist for iMIND Research Study.
(DOCX)

S4 Study Protocol. IRB protocol application and study outline for this manuscript’s timeline submitted for the iMIND Research Study.
(DOCX)
