# Supplementary material for: Retinal layer structure on optical coherence tomography in post-traumatic stress disorder
Source: PLoS One. 2026 Oct 27;21(10):e0360471. doi: 10.1371/journal.pone.0360471 (PMC13712591; doi:10.1371/journal.pone.0360471)
Supplement: Supplementary file 1 [file pone.0360471.s001.docx]

**Appendix A.**

**Retinal Layer**

| **Variable** | **Statistic** | **Control** | **PTSD** | **P-value*** |
| --- | --- | --- | --- | --- |
| retinal center point thickness | N | 81 | 81 |  |
|  | Mean (SD) | 237.497 (25.100) | 229.604 (21.722) | 0.230 |
|  | Min, Median, Max | 195.313, 231.445, 337.891 | 177.734, 226.486, 298.828 |  |
| retinal center 1-mm thickness | N | 81 | 81 |  |
|  | Mean (SD) | 280.587 (23.388) | 273.830 (21.262) | 0.220 |
|  | Min, Median, Max | 222.550, 277.531, 379.953 | 215.927, 270.707, 326.141 |  |
| retinal nasal 3-mm thickness | N | 81 | 81 |  |
|  | Mean (SD) | 343.866 (17.382) | 342.113 (16.705) | 0.566 |
|  | Min, Median, Max | 308.024, 338.123, 411.758 | 309.619, 342.640, 381.588 |  |
| retinal superior 3-mm thickness | N | 81 | 81 |  |
|  | Mean (SD) | 342.460 (17.700) | 339.804 (16.126) | 0.409 |
|  | Min, Median, Max | 310.669, 340.684, 399.709 | 302.095, 339.201, 372.555 |  |
| retinal temporal 3-mm thickness | N | 81 | 81 |  |
|  | Mean (SD) | 329.341 (15.481) | 326.865 (14.776) | 0.518 |
|  | Min, Median, Max | 290.877, 325.704, 379.245 | 295.735, 327.136, 358.690 |  |
| retinal inferior 3-mm thickness | N | 81 | 81 |  |
|  | Mean (SD) | 337.090 (17.119) | 334.444 (16.893) | 0.475 |
|  | Min, Median, Max | 295.106, 334.216, 390.315 | 292.201, 335.804, 372.528 |  |
| retinal nasal 6-mm thickness | N | 81 | 81 |  |
|  | Mean (SD) | 312.695 (18.707) | 310.482 (17.128) | 0.186 |
|  | Min, Median, Max | 276.112, 311.824, 361.905 | 272.602, 309.572, 347.601 |  |
| retinal superior 6-mm thickness | N | 81 | 81 |  |
|  | Mean (SD) | 293.889 (15.948) | 291.240 (13.979) | 0.403 |
|  | Min, Median, Max | 268.095, 291.333, 335.954 | 259.338, 290.045, 316.518 |  |
| retinal temporal 6-mm thickness | N | 81 | 81 |  |
|  | Mean (SD) | 280.369 (15.339) | 277.550 (13.319) | 0.578 |
|  | Min, Median, Max | 246.778, 276.572, 320.419 | 243.576, 274.934, 308.342 |  |
| retinal inferior 6-mm thickness | N | 81 | 81 |  |
|  | Mean (SD) | 283.690 (16.149) | 280.002 (14.990) | 0.460 |
|  | Min, Median, Max | 252.673, 282.549, 319.869 | 242.329, 278.691, 314.942 |  |
| retinal center 1-mm volume | N | 81 | 81 |  |
|  | Mean (SD) | 0.220 (0.018) | 0.215 (0.017) | 0.220 |
|  | Min, Median, Max | 0.175, 0.218, 0.298 | 0.170, 0.213, 0.256 |  |
| retinal nasal 3-mm volume | N | 81 | 81 |  |
|  | Mean (SD) | 0.540 (0.027) | 0.537 (0.026) | 0.566 |
|  | Min, Median, Max | 0.484, 0.531, 0.647 | 0.486, 0.538, 0.599 |  |
| retinal superior 3-mm volume | N | 81 | 81 |  |
|  | Mean (SD) | 0.538 (0.028) | 0.534 (0.025) | 0.409 |
|  | Min, Median, Max | 0.488, 0.535, 0.628 | 0.475, 0.533, 0.585 |  |
| retinal temporal 3-mm volume | N | 81 | 81 |  |
|  | Mean (SD) | 0.517 (0.024) | 0.513 (0.023) | 0.518 |
|  | Min, Median, Max | 0.457, 0.512, 0.596 | 0.465, 0.514, 0.563 |  |
| retinal inferior 3-mm volume | N | 81 | 81 |  |
|  | Mean (SD) | 0.530 (0.027) | 0.525 (0.027) | 0.475 |
|  | Min, Median, Max | 0.464, 0.525, 0.613 | 0.459, 0.527, 0.585 |  |
| retinal nasal 6-mm volume | N | 81 | 81 |  |
|  | Mean (SD) | 1.658 (0.099) | 1.646 (0.091) | 0.186 |
|  | Min, Median, Max | 1.464, 1.653, 1.919 | 1.445, 1.641, 1.843 |  |
| retinal superior 6-mm volume | N | 81 | 81 |  |
|  | Mean (SD) | 1.558 (0.085) | 1.544 (0.074) | 0.403 |
|  | Min, Median, Max | 1.421, 1.544, 1.781 | 1.375, 1.538, 1.678 |  |
| retinal temporal 6-mm volume | N | 81 | 81 |  |
|  | Mean (SD) | 1.486 (0.081) | 1.471 (0.071) | 0.578 |
|  | Min, Median, Max | 1.308, 1.466, 1.699 | 1.291, 1.458, 1.635 |  |
| retinal inferior 6-mm volume | N | 81 | 81 |  |
|  | Mean (SD) | 1.504 (0.086) | 1.484 (0.079) | 0.460 |
|  | Min, Median, Max | 1.340, 1.498, 1.696 | 1.285, 1.477, 1.670 |  |
| **P-value based on Z-test of difference between group means using generalized estimating equations (GEE) to account for the correlations between eyes of the same patient.* | | | | |

**Ganglion Cell-inner Plexiform Layer**

| **Variable** | **Statistic** | **Control** | **PTSD** | **P-value*** |
| --- | --- | --- | --- | --- |
| ganglion cell-inner plexiform layer center point thickness | N | 81 | 81 |  |
|  | Mean (SD) | 7.286 (14.927) | 4.332 (8.732) | 0.131 |
|  | Min, Median, Max | 0.000, 0.000, 78.125 | 0.000, 0.000, 46.875 |  |
| ganglion cell-inner plexiform layer center 1-mm thickness | N | 81 | 81 |  |
|  | Mean (SD) | 39.366 (13.204) | 35.499 (10.859) | 0.076 |
|  | Min, Median, Max | 14.408, 37.917, 87.005 | 13.641, 33.738, 65.519 |  |
| ganglion cell-inner plexiform layer nasal 3-mm thickness | N | 81 | 81 |  |
|  | Mean (SD) | 93.226 (10.371) | 93.578 (9.125) | 0.959 |
|  | Min, Median, Max | 67.286, 93.378, 127.668 | 74.279, 92.208, 120.186 |  |
| ganglion cell-inner plexiform layer superior 3-mm thickness | N | 81 | 81 |  |
|  | Mean (SD) | 94.100 (9.874) | 93.013 (8.344) | 0.544 |
|  | Min, Median, Max | 74.790, 94.520, 120.892 | 62.240, 92.242, 112.103 |  |
| ganglion cell-inner plexiform layer temporal 3-mm thickness | N | 81 | 81 |  |
|  | Mean (SD) | 89.126 (9.203) | 88.035 (7.835) | 0.646 |
|  | Min, Median, Max | 70.715, 89.022, 113.622 | 65.992, 87.805, 104.757 |  |
| ganglion cell-inner plexiform layer inferior 3-mm thickness | N | 81 | 81 |  |
|  | Mean (SD) | 93.258 (10.501) | 92.191 (8.778) | 0.549 |
|  | Min, Median, Max | 69.693, 92.983, 122.184 | 61.948, 92.086, 113.378 |  |
| ganglion cell-inner plexiform layer nasal 6-mm thickness | N | 81 | 81 |  |
|  | Mean (SD) | 67.536 (7.304) | 68.069 (6.176) | 0.568 |
|  | Min, Median, Max | 53.805, 66.311, 87.597 | 56.429, 66.751, 86.396 |  |
| ganglion cell-inner plexiform layer superior 6-mm thickness | N | 81 | 81 |  |
|  | Mean (SD) | 60.539 (5.757) | 60.368 (5.497) | 0.865 |
|  | Min, Median, Max | 48.652, 60.359, 78.792 | 48.159, 60.337, 69.843 |  |
| ganglion cell-inner plexiform layer temporal 6-mm thickness | N | 81 | 81 |  |
|  | Mean (SD) | 67.283 (7.530) | 65.317 (5.797) | 0.133 |
|  | Min, Median, Max | 49.891, 65.746, 86.046 | 50.469, 64.519, 78.442 |  |
| ganglion cell-inner plexiform layer inferior 6-mm thickness | N | 81 | 81 |  |
|  | Mean (SD) | 59.688 (6.199) | 58.494 (5.729) | 0.306 |
|  | Min, Median, Max | 47.208, 59.808, 76.523 | 47.902, 58.369, 75.588 |  |
| ganglion cell-inner plexiform layer center 1-mm volume | N | 81 | 81 |  |
|  | Mean (SD) | 0.031 (0.010) | 0.028 (0.009) | 0.076 |
|  | Min, Median, Max | 0.011, 0.029, 0.068 | 0.011, 0.026, 0.051 |  |
| ganglion cell-inner plexiform layer nasal 3-mm volume | N | 81 | 81 |  |
|  | Mean (SD) | 0.146 (0.016) | 0.147 (0.014) | 0.959 |
|  | Min, Median, Max | 0.106, 0.147, 0.201 | 0.117, 0.145, 0.189 |  |
| ganglion cell-inner plexiform layer superior 3-mm volume | N | 81 | 81 |  |
|  | Mean (SD) | 0.148 (0.016) | 0.146 (0.013) | 0.544 |
|  | Min, Median, Max | 0.117, 0.148, 0.190 | 0.098, 0.145, 0.176 |  |
| ganglion cell-inner plexiform layer temporal 3-mm volume | N | 81 | 81 |  |
|  | Mean (SD) | 0.140 (0.015) | 0.138 (0.012) | 0.677 |
|  | Min, Median, Max | 0.111, 0.140, 0.178 | 0.104, 0.138, 0.165 |  |
| ganglion cell-inner plexiform layer inferior 3-mm volume | N | 81 | 81 |  |
|  | Mean (SD) | 0.146 (0.016) | 0.145 (0.014) | 0.549 |
|  | Min, Median, Max | 0.109, 0.146, 0.192 | 0.097, 0.145, 0.178 |  |
| ganglion cell-inner plexiform layer nasal 6-mm volume | N | 81 | 81 |  |
|  | Mean (SD) | 0.358 (0.039) | 0.361 (0.033) | 0.568 |
|  | Min, Median, Max | 0.285, 0.352, 0.464 | 0.299, 0.354, 0.458 |  |
| ganglion cell-inner plexiform layer superior 6-mm volume | N | 81 | 81 |  |
|  | Mean (SD) | 0.321 (0.031) | 0.320 (0.029) | 0.865 |
|  | Min, Median, Max | 0.258, 0.320, 0.418 | 0.255, 0.320, 0.370 |  |
| ganglion cell-inner plexiform layer temporal 6-mm volume | N | 81 | 81 |  |
|  | Mean (SD) | 0.357 (0.040) | 0.346 (0.031) | 0.133 |
|  | Min, Median, Max | 0.264, 0.349, 0.456 | 0.268, 0.342, 0.416 |  |
| ganglion cell-inner plexiform layer inferior 6-mm volume | N | 81 | 81 |  |
|  | Mean (SD) | 0.316 (0.033) | 0.310 (0.030) | 0.306 |
|  | Min, Median, Max | 0.250, 0.317, 0.406 | 0.254, 0.309, 0.401 |  |
| **P-value based on Z-test of difference between group means using generalized estimating equations (GEE) to account for the correlations between eyes of the same patient* | | | | |

**Inner Nuclear Layer**

| **Variable** | **Statistic** | **Control** | **PTSD** | **P-value*** |
| --- | --- | --- | --- | --- |
| inner nuclear layer center point thickness | N | 81 | 81 |  |
|  | Mean (SD) | 6.634 (8.028) | 5.300 (8.094) | 0.599 |
|  | Min, Median, Max | 0.000, 3.906, 37.109 | 0.000, 1.953, 37.109 |  |
| inner nuclear layer center 1-mm thickness | N | 81 | 81 |  |
|  | Mean (SD) | 21.478 (4.555) | 20.833 (5.450) | 0.209 |
|  | Min, Median, Max | 11.686, 20.789, 35.305 | 12.656, 19.503, 38.249 |  |
| inner nuclear layer nasal 3-mm thickness | N | 81 | 81 |  |
|  | Mean (SD) | 39.202 (2.120) | 39.719 (2.418) | 0.164 |
|  | Min, Median, Max | 33.607, 39.126, 43.409 | 32.425, 40.046, 44.193 |  |
| inner nuclear layer superior 3-mm thickness | N | 81 | 81 |  |
|  | Mean (SD) | 39.522 (2.327) | 39.868 (2.229) | 0.431 |
|  | Min, Median, Max | 33.678, 39.957, 44.151 | 35.113, 39.941, 44.249 |  |
| inner nuclear layer temporal 3-mm thickness | N | 81 | 81 |  |
|  | Mean (SD) | 37.061 (2.682) | 37.161 (2.534) | 0.699 |
|  | Min, Median, Max | 31.419, 37.046, 42.140 | 31.991, 36.995, 41.664 |  |
| inner nuclear layer inferior 3-mm thickness | N | 81 | 81 |  |
|  | Mean (SD) | 39.656 (2.545) | 39.866 (2.366) | 0.681 |
|  | Min, Median, Max | 31.560, 40.036, 44.183 | 30.000, 40.107, 44.245 |  |
| inner nuclear layer nasal 6-mm thickness | N | 81 | 81 |  |
|  | Mean (SD) | 31.978 (2.559) | 32.420 (2.494) | 0.306 |
|  | Min, Median, Max | 28.114, 31.408, 39.033 | 27.705, 31.901, 37.976 |  |
| inner nuclear layer superior 6-mm thickness | N | 81 | 81 |  |
|  | Mean (SD) | 28.867 (2.490) | 29.297 (2.426) | 0.415 |
|  | Min, Median, Max | 23.870, 28.290, 37.778 | 24.905, 29.123, 34.342 |  |
| inner nuclear layer temporal 6-mm thickness | N | 81 | 81 |  |
|  | Mean (SD) | 31.360 (2.635) | 31.225 (2.235) | 0.642 |
|  | Min, Median, Max | 26.524, 30.644, 38.580 | 27.100, 30.838, 37.313 |  |
| inner nuclear layer inferior 6-mm thickness | N | 81 | 81 |  |
|  | Mean (SD) | 28.915 (2.498) | 28.842 (2.353) | 0.914 |
|  | Min, Median, Max | 23.631, 28.673, 36.239 | 24.410, 28.586, 34.597 |  |
| inner nuclear layer center 1-mm volume | N | 81 | 81 |  |
|  | Mean (SD) | 0.017 (0.004) | 0.016 (0.004) | 0.209 |
|  | Min, Median, Max | 0.009, 0.016, 0.028 | 0.010, 0.015, 0.030 |  |
| inner nuclear layer nasal 3-mm volume | N | 81 | 81 |  |
|  | Mean (SD) | 0.062 (0.003) | 0.062 (0.004) | 0.164 |
|  | Min, Median, Max | 0.053, 0.061, 0.068 | 0.051, 0.063, 0.069 |  |
| inner nuclear layer superior 3-mm volume | N | 81 | 81 |  |
|  | Mean (SD) | 0.062 (0.004) | 0.063 (0.004) | 0.431 |
|  | Min, Median, Max | 0.053, 0.063, 0.069 | 0.055, 0.063, 0.070 |  |
| inner nuclear layer temporal 3-mm volume | N | 81 | 81 |  |
|  | Mean (SD) | 0.058 (0.004) | 0.058 (0.004) | 0.689 |
|  | Min, Median, Max | 0.049, 0.058, 0.066 | 0.050, 0.058, 0.065 |  |
| inner nuclear layer inferior 3-mm volume | N | 81 | 81 |  |
|  | Mean (SD) | 0.062 (0.004) | 0.063 (0.004) | 0.681 |
|  | Min, Median, Max | 0.050, 0.063, 0.069 | 0.047, 0.063, 0.069 |  |
| inner nuclear layer nasal 6-mm volume | N | 81 | 81 |  |
|  | Mean (SD) | 0.170 (0.014) | 0.172 (0.013) | 0.306 |
|  | Min, Median, Max | 0.149, 0.167, 0.207 | 0.147, 0.169, 0.201 |  |
| inner nuclear layer superior 6-mm volume | N | 81 | 81 |  |
|  | Mean (SD) | 0.153 (0.013) | 0.155 (0.013) | 0.415 |
|  | Min, Median, Max | 0.127, 0.150, 0.200 | 0.132, 0.154, 0.182 |  |
| inner nuclear layer temporal 6-mm volume | N | 81 | 81 |  |
|  | Mean (SD) | 0.166 (0.014) | 0.166 (0.012) | 0.642 |
|  | Min, Median, Max | 0.141, 0.162, 0.205 | 0.144, 0.163, 0.198 |  |
| inner nuclear layer inferior 6-mm volume | N | 81 | 81 |  |
|  | Mean (SD) | 0.153 (0.013) | 0.153 (0.012) | 0.914 |
|  | Min, Median, Max | 0.125, 0.152, 0.192 | 0.129, 0.152, 0.183 |  |
| **P-value based on Z-test of difference between group means using generalized estimating equations (GEE) to account for the correlations between eyes of the same patient* | | | | |

**Outer Plexiform Layer**

| **Variable** | **Statistic** | **Control** | **PTSD** | **P-value*** |
| --- | --- | --- | --- | --- |
| outer plexiform layer center point thickness | N | 81 | 81 |  |
|  | Mean (SD) | 6.046 (7.396) | 5.602 (8.946) | 0.821 |
|  | Min, Median, Max | 0.000, 1.953, 27.344 | 0.000, 1.953, 44.133 |  |
| outer plexiform layer center 1-mm thickness | N | 81 | 81 |  |
|  | Mean (SD) | 23.420 (5.323) | 21.781 (4.958) | 0.084 |
|  | Min, Median, Max | 11.916, 22.763, 37.689 | 13.810, 21.392, 36.557 |  |
| outer plexiform layer nasal 3-mm thickness | N | 81 | 81 |  |
|  | Mean (SD) | 30.305 (7.141) | 32.617 (9.083) | 0.131 |
|  | Min, Median, Max | 23.442, 28.268, 55.685 | 23.076, 29.130, 57.980 |  |
| outer plexiform layer superior 3-mm thickness | N | 81 | 81 |  |
|  | Mean (SD) | 27.308 (3.384) | 27.117 (3.387) | 0.748 |
|  | Min, Median, Max | 23.342, 26.761, 45.178 | 23.368, 26.261, 43.499 |  |
| outer plexiform layer temporal 3-mm thickness | N | 81 | 81 |  |
|  | Mean (SD) | 26.635 (3.798) | 25.285 (1.355) | **0.013** |
|  | Min, Median, Max | 22.908, 25.809, 49.077 | 23.126, 25.059, 29.295 |  |
| outer plexiform layer inferior 3-mm thickness | N | 81 | 81 |  |
|  | Mean (SD) | 31.568 (7.661) | 30.045 (6.349) | 0.169 |
|  | Min, Median, Max | 23.212, 28.826, 63.065 | 21.273, 27.765, 56.327 |  |
| outer plexiform layer nasal 6-mm thickness | N | 81 | 81 |  |
|  | Mean (SD) | 26.526 (3.161) | 27.173 (3.721) | 0.379 |
|  | Min, Median, Max | 21.748, 25.872, 36.512 | 22.564, 25.837, 36.751 |  |
| outer plexiform layer superior 6-mm thickness | N | 81 | 81 |  |
|  | Mean (SD) | 23.359 (1.380) | 23.181 (1.052) | 0.458 |
|  | Min, Median, Max | 21.448, 23.021, 29.869 | 21.603, 23.016, 26.851 |  |
| outer plexiform layer temporal 6-mm thickness | N | 81 | 81 |  |
|  | Mean (SD) | 23.989 (1.537) | 23.467 (0.884) | **0.026** |
|  | Min, Median, Max | 22.002, 23.631, 29.748 | 22.002, 23.293, 27.045 |  |
| outer plexiform layer inferior 6-mm thickness | N | 81 | 81 |  |
|  | Mean (SD) | 24.775 (2.335) | 23.978 (1.578) | **0.035** |
|  | Min, Median, Max | 21.652, 24.150, 32.813 | 21.559, 23.814, 28.608 |  |
| outer plexiform layer center 1-mm volume | N | 81 | 81 |  |
|  | Mean (SD) | 0.018 (0.004) | 0.017 (0.004) | 0.084 |
|  | Min, Median, Max | 0.009, 0.018, 0.030 | 0.011, 0.017, 0.029 |  |
| outer plexiform layer nasal 3-mm volume | N | 81 | 81 |  |
|  | Mean (SD) | 0.048 (0.011) | 0.051 (0.014) | 0.131 |
|  | Min, Median, Max | 0.037, 0.044, 0.087 | 0.036, 0.046, 0.091 |  |
| outer plexiform layer superior 3-mm volume | N | 81 | 81 |  |
|  | Mean (SD) | 0.043 (0.005) | 0.043 (0.005) | 0.748 |
|  | Min, Median, Max | 0.037, 0.042, 0.071 | 0.037, 0.041, 0.068 |  |
| outer plexiform layer temporal 3-mm volume | N | 81 | 81 |  |
|  | Mean (SD) | 0.042 (0.006) | 0.040 (0.002) | **0.013** |
|  | Min, Median, Max | 0.036, 0.041, 0.077 | 0.036, 0.039, 0.046 |  |
| outer plexiform layer inferior 3-mm volume | N | 81 | 81 |  |
|  | Mean (SD) | 0.050 (0.012) | 0.047 (0.010) | 0.169 |
|  | Min, Median, Max | 0.036, 0.045, 0.099 | 0.033, 0.044, 0.088 |  |
| outer plexiform layer nasal 6-mm volume | N | 81 | 81 |  |
|  | Mean (SD) | 0.141 (0.017) | 0.144 (0.020) | 0.379 |
|  | Min, Median, Max | 0.115, 0.137, 0.194 | 0.120, 0.137, 0.195 |  |
| outer plexiform layer superior 6-mm volume | N | 81 | 81 |  |
|  | Mean (SD) | 0.124 (0.007) | 0.123 (0.006) | 0.458 |
|  | Min, Median, Max | 0.114, 0.122, 0.158 | 0.115, 0.122, 0.142 |  |
| outer plexiform layer temporal 6-mm volume | N | 81 | 81 |  |
|  | Mean (SD) | 0.127 (0.008) | 0.124 (0.005) | **0.026** |
|  | Min, Median, Max | 0.117, 0.125, 0.158 | 0.117, 0.123, 0.143 |  |
| outer plexiform layer inferior 6-mm volume | N | 81 | 81 |  |
|  | Mean (SD) | 0.131 (0.012) | 0.127 (0.008) | **0.035** |
|  | Min, Median, Max | 0.115, 0.128, 0.174 | 0.114, 0.126, 0.152 |  |
| **P-value based on Z-test of difference between group means using generalized estimating equations (GEE) to account for the correlations between eyes of the same patient* | | | | |

**Outer Nuclear Layer**

| **Variable** | **Statistic** | **Control** | **PTSD** | **P-value*** |
| --- | --- | --- | --- | --- |
| outer nuclear layer center point thickness | N | 81 | 81 |  |
|  | Mean (SD) | 107.164 (17.333) | 102.918 (14.668) | 0.100 |
|  | Min, Median, Max | 60.547, 107.764, 152.344 | 70.313, 103.516, 150.391 |  |
| outer nuclear layer center 1-mm thickness | N | 81 | 81 |  |
|  | Mean (SD) | 92.436 (10.459) | 91.236 (10.504) | 0.576 |
|  | Min, Median, Max | 70.367, 93.069, 120.002 | 46.502, 92.929, 114.933 |  |
| outer nuclear layer nasal 3-mm thickness | N | 81 | 81 |  |
|  | Mean (SD) | 74.339 (11.525) | 70.777 (13.307) | 0.168 |
|  | Min, Median, Max | 37.596, 77.110, 93.307 | 38.460, 74.722, 92.765 |  |
| outer nuclear layer superior 3-mm thickness | N | 81 | 81 |  |
|  | Mean (SD) | 74.287 (8.549) | 73.795 (8.583) | 0.096 |
|  | Min, Median, Max | 48.450, 74.584, 91.749 | 52.752, 74.116, 90.371 |  |
| outer nuclear layer temporal 3-mm thickness | N | 81 | 81 |  |
|  | Mean (SD) | 76.614 (8.286) | 76.642 (8.013) | 0.864 |
|  | Min, Median, Max | 49.357, 76.420, 93.666 | 46.471, 76.588, 93.885 |  |
| outer nuclear layer inferior 3-mm thickness | N | 81 | 81 |  |
|  | Mean (SD) | 66.917 (10.476) | 67.073 (11.244) | 0.837 |
|  | Min, Median, Max | 40.471, 68.877, 83.690 | 22.215, 68.257, 85.860 |  |
| outer nuclear layer nasal 6-mm thickness | N | 81 | 81 |  |
|  | Mean (SD) | 56.736 (7.473) | 55.956 (7.901) | **0.039** |
|  | Min, Median, Max | 36.422, 57.260, 71.741 | 36.615, 56.397, 74.660 |  |
| outer nuclear layer superior 6-mm thickness | N | 81 | 81 |  |
|  | Mean (SD) | 62.166 (6.962) | 61.961 (7.407) | 0.338 |
|  | Min, Median, Max | 42.392, 62.544, 77.312 | 42.392, 61.927, 78.428 |  |
| outer nuclear layer temporal 6-mm thickness | N | 81 | 81 |  |
|  | Mean (SD) | 60.427 (6.270) | 60.190 (6.325) | 0.071 |
|  | Min, Median, Max | 47.755, 61.058, 73.024 | 47.755, 60.047, 74.246 |  |
| outer nuclear layer inferior 6-mm thickness | N | 81 | 81 |  |
|  | Mean (SD) | 51.548 (6.562) | 52.396 (7.101) | 0.238 |
|  | Min, Median, Max | 33.012, 52.594, 65.455 | 29.920, 52.765, 66.751 |  |
| outer nuclear layer center 1-mm volume | N | 81 | 81 |  |
|  | Mean (SD) | 0.073 (0.008) | 0.072 (0.008) | 0.576 |
|  | Min, Median, Max | 0.055, 0.073, 0.094 | 0.037, 0.073, 0.090 |  |
| outer nuclear layer nasal 3-mm volume | N | 81 | 81 |  |
|  | Mean (SD) | 0.117 (0.018) | 0.111 (0.021) | 0.168 |
|  | Min, Median, Max | 0.059, 0.121, 0.147 | 0.060, 0.117, 0.146 |  |
| outer nuclear layer superior 3-mm volume | N | 81 | 81 |  |
|  | Mean (SD) | 0.117 (0.013) | 0.116 (0.013) | 0.096 |
|  | Min, Median, Max | 0.076, 0.117, 0.144 | 0.083, 0.116, 0.142 |  |
| outer nuclear layer temporal 3-mm volume | N | 81 | 81 |  |
|  | Mean (SD) | 0.120 (0.013) | 0.120 (0.013) | 0.864 |
|  | Min, Median, Max | 0.078, 0.120, 0.147 | 0.073, 0.120, 0.147 |  |
| outer nuclear layer inferior 3-mm volume | N | 81 | 81 |  |
|  | Mean (SD) | 0.105 (0.016) | 0.105 (0.018) | 0.837 |
|  | Min, Median, Max | 0.064, 0.108, 0.131 | 0.035, 0.107, 0.135 |  |
| outer nuclear layer nasal 6-mm volume | N | 81 | 81 |  |
|  | Mean (SD) | 0.301 (0.040) | 0.297 (0.042) | **0.039** |
|  | Min, Median, Max | 0.193, 0.304, 0.380 | 0.194, 0.299, 0.396 |  |
| outer nuclear layer superior 6-mm volume | N | 81 | 81 |  |
|  | Mean (SD) | 0.330 (0.037) | 0.328 (0.039) | 0.338 |
|  | Min, Median, Max | 0.225, 0.332, 0.410 | 0.225, 0.328, 0.416 |  |
| outer nuclear layer temporal 6-mm volume | N | 81 | 81 |  |
|  | Mean (SD) | 0.320 (0.033) | 0.319 (0.034) | 0.071 |
|  | Min, Median, Max | 0.253, 0.324, 0.387 | 0.253, 0.318, 0.394 |  |
| outer nuclear layer inferior 6-mm volume | N | 81 | 81 |  |
|  | Mean (SD) | 0.273 (0.035) | 0.278 (0.038) | 0.238 |
|  | Min, Median, Max | 0.175, 0.279, 0.347 | 0.159, 0.280, 0.354 |  |
| **P-value based on Z-test of difference between group means using generalized estimating equations (GEE) to account for the correlations between eyes of the same patient* | | | | |

**Photoreceptor Layer**

| **Variable** | | **Statistic** | | **Control** | | **PTSD** | | **P-value*** | |
| --- | --- | --- | --- | --- | --- | --- | --- | --- | --- |
| photoreceptors center point thickness | N | | 81 | | 81 | |  | |  |
|  | Mean (SD) | | 69.083 (10.276) | | 64.786 (8.622) | | **0.010** | |  |
|  | Min, Median, Max | | 52.734, 68.359, 109.567 | | 46.875, 63.691, 96.038 | |  | |  |
| photoreceptors center 1-mm thickness | N | | 81 | | 81 | |  | |  |
|  | Mean (SD) | | 62.163 (7.072) | | 57.721 (5.258) | | **0.001** | |  |
|  | Min, Median, Max | | 51.253, 63.549, 74.341 | | 49.490, 56.624, 72.948 | |  | |  |
| photoreceptors nasal 3-mm thickness | N | | 81 | | 81 | |  | |  |
|  | Mean (SD) | | 55.682 (6.611) | | 52.005 (5.139) | | **0.001** | |  |
|  | Min, Median, Max | | 44.933, 56.488, 68.990 | | 42.852, 51.558, 66.723 | |  | |  |
| photoreceptors superior 3-mm thickness | N | | 81 | | 81 | |  | |  |
|  | Mean (SD) | | 54.398 (5.488) | | 50.486 (4.875) | | **<0.001** | |  |
|  | Min, Median, Max | | 45.134, 54.461, 65.911 | | 42.813, 50.252, 66.145 | |  | |  |
| photoreceptors temporal 3-mm thickness | N | | 81 | | 81 | |  | |  |
|  | Mean (SD) | | 55.060 (5.665) | | 50.894 (5.008) | | **<0.001** | |  |
|  | Min, Median, Max | | 44.050, 55.009, 64.700 | | 42.024, 50.533, 66.079 | |  | |  |
| photoreceptors inferior 3-mm thickness | N | | 81 | | 81 | |  | |  |
|  | Mean (SD) | | 53.402 (5.985) | | 49.761 (4.433) | | **0.001** | |  |
|  | Min, Median, Max | | 43.504, 53.929, 66.546 | | 41.990, 49.434, 61.996 | |  | |  |
| photoreceptors nasal 6-mm thickness | N | | 81 | | 81 | |  | |  |
|  | Mean (SD) | | 53.325 (4.868) | | 50.823 (4.042) | | **0.004** | |  |
|  | Min, Median, Max | | 44.957, 53.161, 65.208 | | 43.680, 50.207, 60.610 | |  | |  |
| photoreceptors superior 6-mm thickness | N | | 81 | | 81 | |  | |  |
|  | Mean (SD) | | 54.117 (4.272) | | 52.139 (4.207) | | **0.012** | |  |
|  | Min, Median, Max | | 45.198, 54.190, 63.091 | | 42.795, 52.310, 61.333 | |  | |  |
| photoreceptors temporal 6-mm thickness | N | | 81 | | 81 | |  | |  |
|  | Mean (SD) | | 52.778 (4.349) | | 50.146 (3.943) | | **0.005** | |  |
|  | Min, Median, Max | | 43.747, 52.870, 62.184 | | 42.510, 50.071, 58.605 | |  | |  |
| photoreceptors inferior 6-mm thickness | N | | 81 | | 81 | |  | |  |
|  | Mean (SD) | | 53.011 (3.875) | | 51.307 (3.277) | | **0.008** | |  |
|  | Min, Median, Max | | 44.645, 52.866, 61.861 | | 45.302, 51.358, 58.044 | |  | |  |
| photoreceptors center 1-mm volume | N | | 81 | | 81 | |  | |  |
|  | Mean (SD) | | 0.049 (0.006) | | 0.045 (0.004) | | **0.001** | |  |
|  | Min, Median, Max | | 0.040, 0.050, 0.058 | | 0.039, 0.044, 0.057 | |  | |  |
| photoreceptors nasal 3-mm volume | N | | 81 | | 81 | |  | |  |
|  | Mean (SD) | | 0.087 (0.010) | | 0.082 (0.008) | | **0.001** | |  |
|  | Min, Median, Max | | 0.071, 0.089, 0.108 | | 0.067, 0.081, 0.105 | |  | |  |
| photoreceptors superior 3-mm volume | N | | 81 | | 81 | |  | |  |
|  | Mean (SD) | | 0.085 (0.009) | | 0.079 (0.008) | | **<0.001** | |  |
|  | Min, Median, Max | | 0.071, 0.086, 0.104 | | 0.067, 0.079, 0.104 | |  | |  |
| photoreceptors temporal 3-mm volume | N | | 81 | | 81 | |  | |  |
|  | Mean (SD) | | 0.086 (0.009) | | 0.080 (0.008) | | **<0.001** | |  |
|  | Min, Median, Max | | 0.069, 0.086, 0.102 | | 0.066, 0.079, 0.104 | |  | |  |
| photoreceptors inferior 3-mm volume | N | | 81 | | 81 | |  | |  |
|  | Mean (SD) | | 0.084 (0.009) | | 0.078 (0.007) | | **0.001** | |  |
|  | Min, Median, Max | | 0.068, 0.085, 0.105 | | 0.066, 0.078, 0.097 | |  | |  |
| photoreceptors nasal 6-mm volume | N | | 81 | | 81 | |  | |  |
|  | Mean (SD) | | 0.283 (0.026) | | 0.269 (0.021) | | **0.004** | |  |
|  | Min, Median, Max | | 0.238, 0.282, 0.346 | | 0.232, 0.266, 0.321 | |  | |  |
| photoreceptors superior 6-mm volume | N | | 81 | | 81 | |  | |  |
|  | Mean (SD) | | 0.287 (0.023) | | 0.276 (0.022) | | **0.012** | |  |
|  | Min, Median, Max | | 0.240, 0.287, 0.334 | | 0.227, 0.277, 0.325 | |  | |  |
| photoreceptors temporal 6-mm volume | N | | 81 | | 81 | |  | |  |
|  | Mean (SD) | | 0.280 (0.023) | | 0.266 (0.021) | | **0.005** | |  |
|  | Min, Median, Max | | 0.232, 0.280, 0.330 | | 0.225, 0.265, 0.311 | |  | |  |
| photoreceptors inferior 6-mm volume | N | | 81 | | 81 | |  | |  |
|  | Mean (SD) | | 0.281 (0.021) | | 0.272 (0.017) | | **0.008** | |  |
|  | Min, Median, Max | | 0.237, 0.280, 0.328 | | 0.240, 0.272, 0.308 | |  | |  |
| **P-value based on Z-test of difference between group means using generalized estimating equations (GEE) to account for the correlations between eyes of the same patient* | | | | | | | | |  |

**Retinal Pigment Epithelium**

| **Variable** | **Statistic** | **Control** | **PTSD** | **P-value*** |
| --- | --- | --- | --- | --- |
| retinal pigment epithelium center point thickness | N | 81 | 81 |  |
|  | Mean (SD) | 23.761 (8.627) | 29.632 (5.183) | **<0.001** |
|  | Min, Median, Max | 10.275, 21.484, 44.073 | 11.621, 30.450, 40.908 |  |
| retinal pigment epithelium center 1-mm thickness | N | 81 | 81 |  |
|  | Mean (SD) | 24.102 (6.088) | 29.178 (3.872) | **<0.001** |
|  | Min, Median, Max | 14.665, 24.883, 34.001 | 16.924, 30.038, 33.372 |  |
| retinal pigment epithelium nasal 3-mm thickness | N | 81 | 81 |  |
|  | Mean (SD) | 23.819 (5.217) | 27.384 (3.849) | **<0.001** |
|  | Min, Median, Max | 16.368, 23.432, 32.615 | 16.585, 27.704, 33.520 |  |
| retinal pigment epithelium superior 3-mm thickness | N | 81 | 81 |  |
|  | Mean (SD) | 23.504 (4.302) | 26.958 (3.650) | **<0.001** |
|  | Min, Median, Max | 15.902, 23.231, 32.868 | 17.239, 27.242, 33.457 |  |
| retinal pigment epithelium temporal 3-mm thickness | N | 81 | 81 |  |
|  | Mean (SD) | 23.420 (4.655) | 27.399 (3.768) | **<0.001** |
|  | Min, Median, Max | 15.942, 22.445, 32.240 | 17.247, 27.278, 32.994 |  |
| retinal pigment epithelium inferior 3-mm thickness | N | 81 | 81 |  |
|  | Mean (SD) | 23.567 (4.447) | 26.871 (3.515) | **<0.001** |
|  | Min, Median, Max | 17.254, 22.407, 32.505 | 17.759, 26.775, 33.344 |  |
| retinal pigment epithelium nasal 6-mm thickness | N | 81 | 81 |  |
|  | Mean (SD) | 21.751 (3.480) | 24.733 (3.298) | **<0.001** |
|  | Min, Median, Max | 14.719, 21.262, 30.794 | 16.602, 24.261, 31.724 |  |
| retinal pigment epithelium superior 6-mm thickness | N | 81 | 81 |  |
|  | Mean (SD) | 22.175 (2.837) | 24.558 (3.412) | **<0.001** |
|  | Min, Median, Max | 16.096, 22.422, 30.202 | 18.265, 24.037, 33.057 |  |
| retinal pigment epithelium temporal 6-mm thickness | N | 81 | 81 |  |
|  | Mean (SD) | 21.842 (3.062) | 24.848 (3.339) | **<0.001** |
|  | Min, Median, Max | 14.804, 21.729, 30.514 | 18.113, 24.594, 32.959 |  |
| retinal pigment epithelium inferior 6-mm thickness | N | 81 | 81 |  |
|  | Mean (SD) | 21.414 (2.334) | 23.572 (2.761) | **<0.001** |
|  | Min, Median, Max | 16.284, 20.993, 28.680 | 18.591, 23.171, 30.441 |  |
| retinal pigment epithelium center 1-mm volume | N | 81 | 81 |  |
|  | Mean (SD) | 0.019 (0.005) | 0.023 (0.003) | **<0.001** |
|  | Min, Median, Max | 0.012, 0.020, 0.027 | 0.013, 0.024, 0.026 |  |
| retinal pigment epithelium nasal 3-mm volume | N | 81 | 81 |  |
|  | Mean (SD) | 0.037 (0.008) | 0.043 (0.006) | **<0.001** |
|  | Min, Median, Max | 0.026, 0.037, 0.051 | 0.026, 0.044, 0.053 |  |
| retinal pigment epithelium superior 3-mm volume | N | 81 | 81 |  |
|  | Mean (SD) | 0.037 (0.007) | 0.042 (0.006) | **<0.001** |
|  | Min, Median, Max | 0.025, 0.036, 0.052 | 0.027, 0.043, 0.053 |  |
| retinal pigment epithelium temporal 3-mm volume | N | 81 | 81 |  |
|  | Mean (SD) | 0.037 (0.007) | 0.043 (0.006) | **<0.001** |
|  | Min, Median, Max | 0.025, 0.035, 0.051 | 0.027, 0.043, 0.052 |  |
| retinal pigment epithelium inferior 3-mm volume | N | 81 | 81 |  |
|  | Mean (SD) | 0.037 (0.007) | 0.042 (0.006) | **<0.001** |
|  | Min, Median, Max | 0.027, 0.035, 0.051 | 0.028, 0.042, 0.052 |  |
| retinal pigment epithelium nasal 6-mm volume | N | 81 | 81 |  |
|  | Mean (SD) | 0.115 (0.018) | 0.131 (0.017) | **<0.001** |
|  | Min, Median, Max | 0.078, 0.113, 0.163 | 0.088, 0.129, 0.168 |  |
| retinal pigment epithelium superior 6-mm volume | N | 81 | 81 |  |
|  | Mean (SD) | 0.118 (0.015) | 0.130 (0.018) | **<0.001** |
|  | Min, Median, Max | 0.085, 0.119, 0.160 | 0.097, 0.127, 0.175 |  |
| retinal pigment epithelium temporal 6-mm volume | N | 81 | 81 |  |
|  | Mean (SD) | 0.116 (0.016) | 0.132 (0.018) | **<0.001** |
|  | Min, Median, Max | 0.078, 0.115, 0.162 | 0.096, 0.130, 0.175 |  |
| retinal pigment epithelium inferior 6-mm volume | N | 81 | 81 |  |
|  | Mean (SD) | 0.114 (0.012) | 0.125 (0.015) | **<0.001** |
|  | Min, Median, Max | 0.086, 0.111, 0.152 | 0.099, 0.123, 0.161 |  |
| **P-value based on Z-test of difference between group means using generalized estimating equations (GEE) to account for the correlations between eyes of the same patient* | | | | |
