# Supplementary material for: Retinal layer structure on optical coherence tomography in post-traumatic stress disorder
Source: PLoS One. 2026 Oct 27;21(10):e0360471. doi: 10.1371/journal.pone.0360471 (PMC13712591; doi:10.1371/journal.pone.0360471)
Supplement: Supplementary file 2 [file pone.0360471.s002.docx]

**Appendix B.**

**Magnitude of Significant Retinal Layer Differences**

**Outer Plexiform Layer**

| **Variable** | **Control Mean (SD)** | **PTSD Mean (SD)** | **Absolute Difference** | **Percentage Difference (%)** | **P-value†** |
| --- | --- | --- | --- | --- | --- |
| outer plexiform layer temporal 3-mm thickness | 26.635 (3.798) | 25.285 (1.355) | 1.350 | 5.1 | 0.013 |
| outer plexiform layer temporal 6-mm thickness | 23.989 (1.537) | 23.467 (0.884) | 0.522 | 2.2 | 0.026 |
| outer plexiform layer inferior 6-mm thickness | 24.775 (2.335) | 23.978 (1.578) | 0.797 | 3.2 | 0.035 |
| outer plexiform layer temporal 3-mm volume | 0.042 (0.006) | 0.040 (0.002) | 0.002 | 4.8 | 0.013 |
| outer plexiform layer temporal 6-mm volume | 0.127 (0.008) | 0.124 (0.005) | 0.003 | 2.4 | 0.026 |
| outer plexiform layer inferior 6-mm volume | 0.131 (0.012) | 0.127 (0.008) | 0.004 | 3.1 | 0.035 |

*†P-value based on Z-test of difference between group means using generalized estimating equations (GEE) to account for the correlations between eyes of the same patient.*

*^*^Indicates findings that remained statistically significant after Bonferroni correction (adjusted significance threshold, P < 0.0026). Absolute difference = |PTSD mean - Control mean|. Percentage difference = (absolute difference / Control mean) x 100. Only comparisons with unadjusted P < 0.05 are included.*

**Outer Nuclear Layer**

| **Variable** | **Control Mean (SD)** | **PTSD Mean (SD)** | **Absolute Difference** | **Percentage Difference (%)** | **P-value†** |
| --- | --- | --- | --- | --- | --- |
| outer nuclear layer nasal 6-mm thickness | 56.736 (7.473) | 55.956 (7.901) | 0.780 | 1.4 | 0.039 |
| outer nuclear layer nasal 6-mm volume | 0.301 (0.040) | 0.297 (0.042) | 0.004 | 1.3 | 0.039 |

*†P-value based on Z-test of difference between group means using generalized estimating equations (GEE) to account for the correlations between eyes of the same patient.*

*^*^Indicates findings that remained statistically significant after Bonferroni correction (adjusted significance threshold, P < 0.0026). Absolute difference = |PTSD mean - Control mean|. Percentage difference = (absolute difference / Control mean) x 100. Only comparisons with unadjusted P < 0.05 are included.*

**Photoreceptor Layer**

| **Variable** | **Control Mean (SD)** | **PTSD Mean (SD)** | **Absolute Difference** | **Percentage Difference (%)** | **P-value†** |
| --- | --- | --- | --- | --- | --- |
| photoreceptors center point thickness | 69.083 (10.276) | 64.786 (8.622) | 4.297 | 6.2 | 0.010 |
| photoreceptors center 1-mm thickness | 62.163 (7.072) | 57.721 (5.258) | 4.442 | 7.1 | 0.001* |
| photoreceptors nasal 3-mm thickness | 55.682 (6.611) | 52.005 (5.139) | 3.677 | 6.6 | 0.001* |
| photoreceptors superior 3-mm thickness | 54.398 (5.488) | 50.486 (4.875) | 3.912 | 7.2 | <0.001* |
| photoreceptors temporal 3-mm thickness | 55.060 (5.665) | 50.894 (5.008) | 4.166 | 7.6 | <0.001* |
| photoreceptors inferior 3-mm thickness | 53.402 (5.985) | 49.761 (4.433) | 3.641 | 6.8 | 0.001* |
| photoreceptors nasal 6-mm thickness | 53.325 (4.868) | 50.823 (4.042) | 2.502 | 4.7 | 0.004 |
| photoreceptors superior 6-mm thickness | 54.117 (4.272) | 52.139 (4.207) | 1.978 | 3.7 | 0.012 |
| photoreceptors temporal 6-mm thickness | 52.778 (4.349) | 50.146 (3.943) | 2.632 | 5.0 | 0.005 |
| photoreceptors inferior 6-mm thickness | 53.011 (3.875) | 51.307 (3.277) | 1.704 | 3.2 | 0.008 |
| photoreceptors center 1-mm volume | 0.049 (0.006) | 0.045 (0.004) | 0.004 | 8.2 | 0.001* |
| photoreceptors nasal 3-mm volume | 0.087 (0.010) | 0.082 (0.008) | 0.005 | 5.7 | 0.001* |
| photoreceptors superior 3-mm volume | 0.085 (0.009) | 0.079 (0.008) | 0.006 | 7.1 | <0.001* |
| photoreceptors temporal 3-mm volume | 0.086 (0.009) | 0.080 (0.008) | 0.006 | 7.0 | <0.001* |
| photoreceptors inferior 3-mm volume | 0.084 (0.009) | 0.078 (0.007) | 0.006 | 7.1 | 0.001* |
| photoreceptors nasal 6-mm volume | 0.283 (0.026) | 0.269 (0.021) | 0.014 | 4.9 | 0.004 |
| photoreceptors superior 6-mm volume | 0.287 (0.023) | 0.276 (0.022) | 0.011 | 3.8 | 0.012 |
| photoreceptors temporal 6-mm volume | 0.280 (0.023) | 0.266 (0.021) | 0.014 | 5.0 | 0.005 |
| photoreceptors inferior 6-mm volume | 0.281 (0.021) | 0.272 (0.017) | 0.009 | 3.2 | 0.008 |

*†P-value based on Z-test of difference between group means using generalized estimating equations (GEE) to account for the correlations between eyes of the same patient.*

*^*^Indicates findings that remained statistically significant after Bonferroni correction (adjusted significance threshold, P < 0.0026). Absolute difference = |PTSD mean - Control mean|. Percentage difference = (absolute difference / Control mean) x 100. Only comparisons with unadjusted P < 0.05 are included.*

**Retinal Pigment Epithelium**

| **Variable** | **Control Mean (SD)** | **PTSD Mean (SD)** | **Absolute Difference** | **Percentage Difference (%)** | **P-value†** |
| --- | --- | --- | --- | --- | --- |
| retinal pigment epithelium center point thickness | 23.761 (8.627) | 29.632 (5.183) | 5.871 | 24.7 | <0.001* |
| retinal pigment epithelium center 1-mm thickness | 24.102 (6.088) | 29.178 (3.872) | 5.076 | 21.1 | <0.001* |
| retinal pigment epithelium nasal 3-mm thickness | 23.819 (5.217) | 27.384 (3.849) | 3.565 | 15.0 | <0.001* |
| retinal pigment epithelium superior 3-mm thickness | 23.504 (4.302) | 26.958 (3.650) | 3.454 | 14.7 | <0.001* |
| retinal pigment epithelium temporal 3-mm thickness | 23.420 (4.655) | 27.399 (3.768) | 3.979 | 17.0 | <0.001* |
| retinal pigment epithelium inferior 3-mm thickness | 23.567 (4.447) | 26.871 (3.515) | 3.304 | 14.0 | <0.001* |
| retinal pigment epithelium nasal 6-mm thickness | 21.751 (3.480) | 24.733 (3.298) | 2.982 | 13.7 | <0.001* |
| retinal pigment epithelium superior 6-mm thickness | 22.175 (2.837) | 24.558 (3.412) | 2.383 | 10.7 | <0.001* |
| retinal pigment epithelium temporal 6-mm thickness | 21.842 (3.062) | 24.848 (3.339) | 3.006 | 13.8 | <0.001* |
| retinal pigment epithelium inferior 6-mm thickness | 21.414 (2.334) | 23.572 (2.761) | 2.158 | 10.1 | <0.001* |
| retinal pigment epithelium center 1-mm volume | 0.019 (0.005) | 0.023 (0.003) | 0.004 | 21.1 | <0.001* |
| retinal pigment epithelium nasal 3-mm volume | 0.037 (0.008) | 0.043 (0.006) | 0.006 | 16.2 | <0.001* |
| retinal pigment epithelium superior 3-mm volume | 0.037 (0.007) | 0.042 (0.006) | 0.005 | 13.5 | <0.001* |
| retinal pigment epithelium temporal 3-mm volume | 0.037 (0.007) | 0.043 (0.006) | 0.006 | 16.2 | <0.001* |
| retinal pigment epithelium inferior 3-mm volume | 0.037 (0.007) | 0.042 (0.006) | 0.005 | 13.5 | <0.001* |
| retinal pigment epithelium nasal 6-mm volume | 0.115 (0.018) | 0.131 (0.017) | 0.016 | 13.9 | <0.001* |
| retinal pigment epithelium superior 6-mm volume | 0.118 (0.015) | 0.130 (0.018) | 0.012 | 10.2 | <0.001* |
| retinal pigment epithelium temporal 6-mm volume | 0.116 (0.016) | 0.132 (0.018) | 0.016 | 13.8 | <0.001* |
| retinal pigment epithelium inferior 6-mm volume | 0.114 (0.012) | 0.125 (0.015) | 0.011 | 9.6 | <0.001* |

*†P-value based on Z-test of difference between group means using generalized estimating equations (GEE) to account for the correlations between eyes of the same patient.*

*^*^Indicates findings that remained statistically significant after Bonferroni correction (adjusted significance threshold, P < 0.0026). Absolute difference = |PTSD mean - Control mean|. Percentage difference = (absolute difference / Control mean) x 100. Only comparisons with unadjusted P < 0.05 are included.*
