# Supplementary material for: Retinal layer structure on optical coherence tomography in post-traumatic stress disorder
Source: PLoS One. 2026 Oct 27;21(10):e0360471. doi: 10.1371/journal.pone.0360471 (PMC13712591; doi:10.1371/journal.pone.0360471)
Supplement: Supplementary file 4 [file pone.0360471.s004.docx]

DUHS IRB Application (Version 1.20)

| ***Please enter the full title of your protocol:** | | |
| --- | --- | --- |
| Evaluating the Retinal and Choroidal Microvasculature and Structure using Multimodal Retinal and Choroidal Imaging in Neurodegenerative Disease: iMIND Research Study |  |  |
| ***Please enter the Short Title you would like to use to reference the study:** | | |
| iMIND Research Study  * This field allows you to enter an abbreviated version of the Study Title to quickly identify this study. |  |  |

| **Primary Dept?** | **Department Name** |
| --- | --- |
| ◆ | **DUHS** - Duke Default Department |

| **General Information** | | |
| --- | --- | --- |
|  | | |
| **Add Study Organization(s):** | | |
| **List Study Organizations associated with this protocol:** | |  |
|  |  |  |
| **Assign key study personnel (KSP) access to the protocol** | | |
| *** Please add a Principal Investigator for the study:**  **(Note: Before this study application can be submitted, the PI MUST have completed CITI training)** | |  |
| Fekrat, Sharon |  |  |
| **3.1 If applicable, please select the Key Study personnel: (Note: Before this study application can be submitted, all Key Personnel MUST have completed CITI training)**  *** Denotes roles that are not recognized in OnCore. Please select an appropriate role that is recognized in all clinical research applications (iRIS, OnCore, eREG, etc.)** | |  |
| A) Additional Investigators, Primary Study Coordinator (CRC), and the Primary Regulatory Coordinator (PRC): |  |  |
| Grewal, Dilraj Co-PI |  |  |
| B) All Other Key Personnel |  |  |
| Chang, Isabel Other  Choi, Alex Graduate Student |  |  |

| Corpuz, Elyse Graduate Student Cox, Meredith Graduate Student Cummings, Cameron Graduate Student D'Cunha, Rachel Graduate Student Daniels, Nikki Graduate Student Dasgupta, Anushka Graduate Student Haystead, Alice  Regulatory Coordinator Henao Giraldo, Ricardo Analyst*  Hsu, John Graduate Student Jacumin, Erika Collaborator Johnson, Kim Collaborator Jones, Sarah  Regulatory Coordinator Karthik, Naveen Analyst*  Kimbrough, Dorlan Collaborator  Lee, Jinwon Graduate Student Liu, Andrew Collaborator  Liu, Tina Graduate Student Moore, Kathryn Collaborator Scott, Burton Collaborator Stinnett, Sandra Statistician  Wax, Adam Collaborator Zhao, Wufan Graduate Student Zhu, Michael Graduate Student |  |  |
| --- | --- | --- |
| ***Please add a Study Contact:** | |  |
| Fekrat, Sharon Grewal, Dilraj Haystead, Alice Jones, Sarah |  |  |

| The Study Contact(s) will receive all important system notifications along with the Principal Investigator. (e.g., The study contact(s) are typically the Principal Investigator, Study Coordinator, and Regulatory Coordinator.) |  | |  |
| --- | --- | --- | --- |
| **Oncore** | | | |
| **Please select the Library for your Protocol:** | | | |
| This field is used in OnCore and determines the Reference Lists, Forms, Protocol Annotations, Notifications, and Signoffs available for the protocol. Protocols that require reporting to the NCI (National Cancer Institute), must select the Oncology library.  Oncology  Non-Oncology | |  | |
| **Protocol Application Type** | | | |
| **Select the type of protocol you are creating:** | | | |
| Please see additional criteria and information in the policy titled, “Reliance on the IRB of Another Institution, Organization, or an Independent IRB", on the [**IRB web site**](https://irb.duhs.duke.edu/).   Regular Study Application - Most common. The IRB will determine if the study is eligible for expedited review or requires full board review upon submission.   Application for Exemption from IRB Review - Includes Exempt, Not Human Subject Research, & Not Research.  External IRB Application - Any study using an external IRB as the IRB-of-Record.  Trainee Research While Away from Duke - Research conducted by medical students overseen by the Office of Curriculum & other student/trainee research away from Duke.   Individual Patient Expanded Access, Including Emergency Use - Use of an investigational product under expanded access, including emergency use of an investigational drug or biologic or emergency use of an unapproved device. | |  | |
| **Conflict of Interest** | | | |
| **Are any key personnel an inventor of any of the drugs, devices or technologies used in this research?** | | | |
|  Yes  No | |  | |
| **Do any key personnel have a conflict of interest management plan issued by DOSI-COI related to this research?** | | | |
|  Yes  No | |  | |
| **Oversight Organization Selection** | | | |
| **CRU (Clinical Research Unit) or Oversight Organization Selection:** | | | |
| Please select the CRU.  Ophthalmology  The Clinical Research Unit that takes responsibility for this study.  Please select **Medicine** as the CRU **only** if the PI is in one of these Divisions or Institutes: Endocrinology, Gastroenterology, General Internal Medicine, Geriatrics, Hematology, Infectious Diseases, Nephrology, Pulmonary, Rheumatology & Immunology, Center for Applied Genomics and | |  | |

Precision Medicine, Center for the Study of Aging and Human Development, Duke Molecular Physiology Institute.

Duke

[alexander.richardson@duke.edu](mailto:alexander.richardson@duke.edu)

Student Volunteer

More information on CRUs can be found on the Duke Office of Clinical Research (DOCR) website,

[**http://docr.som.duke.edu**](http://docr.som.duke.edu/)

Questions concerning CRU selection should be directed to [docr.help@dm.duke.edu.](mailto:docr.help@dm.duke.edu)

For questions about the Campus Oversight Organization, please visit [**Campus Oversight**](https://researchinitiatives.duke.edu/programs-services/campus-oversight-organization-duhs-irb-protocols) [**Organization**](https://researchinitiatives.duke.edu/programs-services/campus-oversight-organization-duhs-irb-protocols).

# EXTERNAL KEY PERSONNEL (KP) TOOL

**Use the following External KP Tool to determine each time you consider adding outside Key Personnel. You will need to clear your responses to the following 2 questions each time.**

**List all Key Personnel on the study who are outside Duke if they are engaged in research and relying on the DUHS IRB: (Consider these questions every time you add external key personnel)**

**If you have additional personnel, use the External KP Tool as a guide to determine if the personnel are engaged in research. Only people ENGAGED IN RESEARCH should be added as external key personnel.**

Are the personnel intervening or interacting with the participant?

Yes	No

Are the personnel obtaining or receiving identifiable private information or protected health information (PHI)?

Yes	No

## This person is not engaged. If you have additional personnel to consider, clear your responses and answer the questions again.

If you have answered “no” to the above questions but have external personnel that will handle de-identified or a limited data set of Duke data, and therefore may require an agreement (such as DUA, DTA, visiting scholar or other agreement) and/or accessing PACE, if necessary, please name them and describe what they will be doing in Section 1500 of this application, “Data Management, Analysis and Statistical Considerations” section.

By checking this box, you agree to follow up with ORC and/or DOCR regarding any agreements and that no data will be obtained or transferred until after the appropriate agreements are executed.

**Entry 1**

Alexander Richardon

**Confirm by checking this box, the person you are adding to this section is engaged in the research based upon the two**

**questions above.**

**Name**

**Study Role**

**Email Address**

**Institution / Organization**

**Entry 2**

Boston University School of Medicine

[raj.kundu@duke.edu](mailto:raj.kundu@duke.edu)

Visiting Research Scholar

Raj Kundu

Duke

[spence.cox@duke.edu](mailto:spence.cox@duke.edu)

unpaid undergraduate volunteer

Spence Cox

Duke University

[sejal.d.patel@duke.edu](mailto:sejal.d.patel@duke.edu)

student volunteer

Sejal Patel

**Confirm by checking this box, the person you are adding to this section is engaged in the research based upon the two**

**questions above.**

**Name**

**Study Role**

**Email Address**

**Institution / Organization**

**Entry 3**

**Confirm by checking this box, the person you are adding to this section is engaged in the research based upon the two**

**questions above.**

**Name**

**Study Role**

**Email Address**

**Institution / Organization**

**Entry 4**

**Confirm by checking this box, the person you are adding to this section is engaged in the research based upon the two**

**questions above.**

**Name**

**Study Role**

**Email Address**

**Institution / Organization**

**Entry 5**

**Confirm by checking this box, the person you are adding to this section is engaged in the research based upon the two**

**questions above.**

**Name**

Isabel Chang

**Study Role**

| student volunteer  **Email Address** [isabel.chang@duke.edu](mailto:isabel.chang@duke.edu)  **Institution / Organization** Duke University |  |
| --- | --- |
| **Describe Role of External Personnel:** | |
| Images may be shared with other institutions such as University of Edinburgh in Scotland using their VAMPIRE software and Tan Tock Seng Hospital in Singapore through their password protected COIN software and Queens University in Belfast will be de-identified and shared through DukeBox with a data-sharing/transfer agreement in place. The purpose of image sharing is for analyses of parameters within de-identified study images that Duke does not have the expertise to complete.  Student volunteer Alexander Richardson who is collaborating to develop machine learning models using de-identified images and associated quantitative data using the Duke Compute Cluster.  Duke undergraduate student volunteers Isabel Chang and Spence Cox are student volunteers who work on deidentified image analysis.  Duke undergraduate student volunteer Sejal D Patel will work on deidentified image analysis of all of the iMIND OCT images to obtain retinal thickness layer measurements.  Visiting Research Scholar **Raj Kundu** will collaborate with iMIND to train, test, and deploy machine learning models based on de-identified ophthalmic images and associated quantitative data. Computation (e.g., model training) will be accomplished using the Duke Compute Cluster (DCC) as well as Globus for the transfer of de-identified data to/from the DCC. He will not have access to the code for de-identification, and no data will be shared until a DTA is fully executed. |  |
| **Indicate the Protocol source below:** | |
| The protocol source is the author of the protocol. If the protocol is a joint authorship between multiple sources, select the primary author.  An IRB fee may be assessed for all research that is supported by for-profit entities and requires full board review. For additional information, see the [**IRB fees section of the IRB web site**](https://irb.duhs.duke.edu/about-us/fees).  Duke PI initiated  Commercial / Industry (for-profit entity) initiated Federal Government initiated  Cooperative Group Initiated Foundation (non-profit group) initiated Other |  |
| **Sponsor and Funding Source** | |
| **Add all funding sources for this study: (Select 'Duke University' if you do not have funding or a sponsor for your study).** | |
|  |  |

| **View Details** | **Sponsor Name** | | **Sponsor Type** | **Contract Type:** | **Project Number** | **Award Number** |
| --- | --- | --- | --- | --- | --- | --- |
|  | Department/Division/Faculty  /PI | | Duke |  |  |  |
|  | |  | | | | |

| Sponsor Name: | Department/Division/Faculty/PI | | | | |
| --- | --- | --- | --- | --- | --- |
| Sponsor Type: | Duke | | | | |
| Sponsor Role: | Funding | | | | |
| **Grant/Contract Number:** |  | | | | |
| **Project Period:** | From: | to: |  |  |  |
| **Is Institution the Primary Grant Holder:** | No | | | | |
| **if No, then who is the Primary Grantee?** |  | | | | |
| Contract Type: |  | | | | |
| Project Number: |  | | | | |
| Award Number: |  | | | | |
| Grant Title: |  | | | | |
| PI Name:  (If PI is not the same as identified on the study.) |  | | | | |
| Explain Any Significant Discrepancy: |  | | | | |
|  Optos Inc | Industry | | Drug  /Device Donation |  |  |
| Sponsor Name: | Optos Inc | | | | |
| Sponsor Type: | Industry | | | | |
| Sponsor Role: | Funding | | | | |
| **Grant/Contract Number:** | 393000667 | | | | |
| **Project Period:** | From: to: | | | | |
| **Is Institution the Primary Grant Holder:** | No | | | | |
| **if No, then who is the Primary Grantee?** |  | | | | |
| Contract Type: | Drug/Device Donation | | | | |
| Project Number: |  | | | | |
| Award Number: |  | | | | |
| Grant Title: |  | | | | |
| PI Name:  (If PI is not the same as identified on the study.) |  | | | | |
| Explain Any Significant Discrepancy: |  | | | | |
| VitreoRetinal Surgery Institutional Foundation Research Award  | | |  | 288840 | 383002024 |
| Sponsor Name: | VitreoRetinal Surgery Foundation Research Award | | | | |
| Sponsor Type: | Institutional | | | | |
| Sponsor Role: | Funding | | | | |
| **Grant/Contract Number:** |  | | | | |
| **Project Period:** | From:12/01/2023 | | to:11/30/2024 |  |  |
| **Is Institution the Primary Grant Holder:** | No | | | | |
| **if No, then who is the Primary** |  | | | | |

| **Grantee?** |  | | |
| --- | --- | --- | --- |
| Contract Type: |  | | |
| Project Number: | 288840 | | |
| Award Number: | 383002024 | | |
| Grant Title: | Longitudinal analysis of optical coherence tomography (OCT) and OCT  angiography in patients with multiple sclerosis | | |
| PI Name:  (If PI is not the same as identified on the study.) |  | | |
| Explain Any Significant Discrepancy: |  | | |
| VitreoRetinal Surgery Institutional Foundation Research Award  | |  | 306188 383003603 |
| Sponsor Name: | VitreoRetinal Surgery Foundation Research Award | | |
| Sponsor Type: | Institutional | | |
| Sponsor Role: | Funding | | |
| **Grant/Contract Number:** |  | | |
| **Project Period:** | From:01/14/2026 | to:01/13/2027 |  |
| **Is Institution the Primary Grant Holder:** | No | | |
| **if No, then who is the Primary Grantee?** |  | | |
| Contract Type: |  | | |
| Project Number: | 306188 | | |
| Award Number: | 383003603 | | |
| Grant Title: | Longitudinal Multimodal Retinal and Choroidal Imaging in Cognitively Normal APOE e4 Carriers and Noncarriers: Five-Year Follow Up Findings | | |
| PI Name:  (If PI is not the same as identified on the study.) |  | | |
| Explain Any Significant Discrepancy: |  | | |
| VitreoRetinal Surgery Institutional Foundation Research Award  | |  | 383003631 |
| Sponsor Name: | VitreoRetinal Surgery Foundation Research Award | | |
| Sponsor Type: | Institutional | | |
| Sponsor Role: | Funding | | |
| **Grant/Contract Number:** |  | | |
| **Project Period:** | From:01/14/2026 | to:01/13/2027 |  |
| **Is Institution the Primary Grant Holder:** | No | | |
| **if No, then who is the Primary Grantee?** |  | | |
| Contract Type: |  | | |
| Project Number: |  | | |
| Award Number: | 383003631 | | |
| Grant Title: | Analysis of Retinal Vascular Differences Using Retinal Vascularity Index on Ultra-widefield Fundus Images Along the Alzheimer's | | |

| **Is this a federally funded study?** | |
| --- | --- |
|  Yes  No |  |
| **Does this study have any of the following?**  Industry sponsored protocol Industry funded Duke protocol  Industry funded sub-contract from another institution Industry provided drug/device/biologic  SBIR/STTR funded protocol | |
|  Yes  No |  |
| **As part of this study, will any samples or PHI be transferred to/from Duke to/from anyone other than the Sponsor, a Sponsor subcontractor, or a Funding Source?** | |
|  Yes  No |  |
| **Is the Department of Defense (DOD) a funding source?** | |
|  Yes  No |  |
|  | |
|  |  |
| **Mobile Devices and Software** | |
| **Does this study involve the use of a software or a mobile application?** | |
|  Yes  No  **Please describe the following:**  The developer of the mobile app and how the app will be obtained. What PHI will be collected via the app.  Where the data will be stored and who will have access to it.  The FDA-approved Zeiss imaging camera device is used to obtain the study images but the Cirrus 11.0 analytical software that is installed on that device is investigational. |  |

|  | Continuum |
| --- | --- |
| PI Name:  (If PI is not the same as identified on the study.) |  |
| Explain Any Significant Discrepancy: |  |

| The Optos California Camera and its software are FDA approved.  REDCap is also used for tracking enrollment and for data entry and management  DukeBox is used for de-identified image storage and sharing.  De-identified project data that is mirrored on REDCap is stored as well on Box.  The VAMPIRE (Vessel Assessment and Measurement Platform for Images of the Retina) software will be used to analyze the Optos images. This software application provides efficient, semi-automatic quantification of retinal vessel properties on ultrawidefield fundus camera images. DTA in place with the University of Edinburgh and Optos. [**https://vampire.computing.dundee.ac.uk/index.html**](https://vampire.computing.dundee.ac.uk/index.html)  COIN (Collaborative Ocular Imaging Network) imaging platform software is used for semi-automated choroidal measurements from de-identified OCT images (DTA in place with Tan Tock Seng Hospital, Singapore). [**https://www.ocularimaging.net/home**](https://www.ocularimaging.net/home)  Some of the de-identified images and associated quantitative data will be analyzed using the Duke Compute Cluster (DCC). |  |
| --- | --- |
| **List all software, including third party (non-Duke) and mobile apps, that will be utilized for ascertainment, recruitment, or conduct of the research/project: (eg, MaestroCare, DEDUCE):** | |
| Zeiss Cirrus 11.0 software MaestroCare/Epic DukeBox  VAMPIRE software Duke RedCap  DEDUCE search query will be conducted & used for recruitment Duke PACE  MyChart  COIN Software  DUke Compute Cluster |  |
| **Are you using FCAP (Federated Clinical Analytics Platform) for this study?** | |
|  Yes  No |  |
| **Multi-site Research** | |
| **Is this a multi-site study?** | |
|  |  |

Yes	No

## Complete for each site if Duke is the Primary grant awardee or coordinating center:

**Entry 1**

**Site Name:**

**City: State/Province:**

**Country:**

**Primary Contact Name: Primary Contact Phone: Primary Contact Email:**

**Does the site have an IRB?**

**Site IRB approval expiration**

**date:**

**If date not provided, explanation of why:**

**Has the site granted permission for the research**

**to be conducted?**

**Does the site plan to rely on the DUHS IRB for review?**

**What is the status of the**

**study at this site?**

**Site approval letters or site**

**personnel lists:**

## Site Contact Information

**Site Details**

Yes	No

Yes	No

Yes	No Open

Closed

Attach site approval letters, site closure letters (if applicable), or site personnel lists in the Initial Submission Packet.

# Research Abstract

## Please type your Research Abstract here:

The Research Abstract should summarize the main points of your study in one paragraph. The following guidelines may help you:

1. Purpose and objective (1-2 sentences)
2. Study activities and population group (2-4 sentences)
3. Data analysis and risk/safety issues (1-2 sentences)

| **Purpose and objective:** To develop and evaluate potential non-invasive biomarkers using multimodal retinal and choroidal imaging including optical coherence tomography (OCT), OCT angiography (OCTA), and ultra-widefield (UWF) fundus photography (color photos and autofluorescence photos) to assess retinal and choroidal structure and microvasculature in persons with neurodegenerative conditions including but not limited to mild cognitive impairment (MCI), Parkinson's Disease (PD), Alzheimer’s Disease (AD), and other neurodegenerations such as frontotemporal dementia (FTD), dementia of Lewy bodies, multiple sclerosis, amyotrophic lateral sclerosis (ALS), and Huntington’s disease. Persons with Down syndrome, who are also at risk for cognitive impairment and Alzheimer’s disease, will also be included in addition to traumatic brain injury (TBI) and posttraumatic stress disorder (PTSD), concussion, and normal cognition, among others.  **Study activities and population group:** Cross-sectional, and longitudinal in those individuals able to return, case-control study of at least 3000 participants from Duke Health, including but not limited to the Duke Memory Clinic, Duke Neurology Clinics, Duke Genetics Clinics, Duke Geriatrics Clinics, Duke Primary Care Clinics, etc. Some participants may not have been a patient at Duke but instead are family members or friends of patients at Duke or of study participants who are also interested in participating. Controls with normal cognition will also be enrolled from Duke Health as outlined above.  Corrected distance visual acuity will be obtained in each eye of a study participant. A Mini-Mental State Examination (MMSE) will be administered by trained study staff for each participant. Undilated Zeiss Cirrus optical coherence tomography (OCT) and OCT angiography (OCTA) scans and Optos UWF color and autofluorescence photographs of the retina and optic nerve of each eye of each study participant will be obtained. A 3×3 mm and 6x6 mm scanning area centered on the fovea and a 4.5 x 4.5 mm scanning area centered on the optic disc will be used to capture OCTA images in undilated eyes. Automated software embedded in the Zeiss imaging machine analyzes the OCT and OCTA images and provides quantitative data. De-identified OCT HD-21 images will be analyzed using COIN software by our staff; the COIN software was developed at Tan Tock Seng Hospital in Singapore (DTA in place and included in submission). Undilated Optos fundus photographs will also be captured. Deidentified fundus photographs will be analyzed using the VAMPIRE software ([**http://vampire.computing.dundee.ac.uk/publications.html**](http://vampire.computing.dundee.ac.uk/publications.html)) by study staff with assistance from the University of Edinburgh (DTA and other documents in place and included in submission). These multimodal retinal and optic nerve OCT, OCTA, and UWF images will be used to train, validate, and test machine learning algorithms in collaboration with the Duke Department of Biostatistics and Bioinformatics and the Duke Department of Electrical and Computer Engineering using the Duke Compute Cluster (DCC). De-identified images and quantitative data will be analyzed in DCC. These algorithms are for exploratory research purposes only and will not be used to assess or treat subjects in this study.  **Data analysis and risk/safety issues**: At least 3000 subjects will be recruited. With the assistance of an experienced statistician, we will use a generalized linear model regression as well as other statistical methods to assess how well a given retinal or choroidal imaging biomarker classifies cases vs. controls. ANOVA and generalized estimating equations will be used to determine the significance of differences in retinal microvasculature and structure between and among the study diagnostic groups.  Data collection will be monitored by the study staff on a regular basis. No long-term safety concerns are anticipated as a result of study interventions. Although unlikely, short-term discomfort or side effects to the retinal and choroidal imaging testing will be monitored by study staff and reported to appropriate oversight groups at Duke Health. These events will be monitored by Drs. Grewal and Fekrat and study staff. After-hours contact information will be provided to all study participants in case of unlikely adverse events. |  |
| --- | --- |
| **Research Summary** | |
| **State your primary study objectives** | |
| To evaluate potential retinal and choroidal imaging biomarkers as well as machine learning models using non-invasive OCT and OCTA scans and ultra-widefield color and autofluorescence fundus photographs to assess the retinal microvasculature and structure in persons with neurodegenerative conditions including but not limited to mild cognitive impairment (MCI), Parkinson's Disease (PD), Alzheimer’s Disease (AD), and other neurodegenerations such as frontotemporal dementia (FTD), dementia of Lewy bodies, multiple sclerosis, amyotrophic lateral sclerosis (ALS), and Huntington’s disease. Persons with Down syndrome, who are also at risk for cognitive impairment and Alzheimer’s disease, will also be included in addition to traumatic brain injury (TBI) and posttraumatic stress disorder (PTSD), concussion, and normal cognition, among others. |  |
| **State your secondary study objectives** | |
|  |  |

| N/A |  |
| --- | --- |
| **Please select your research summary form:** | |
| Standard Research Summary Template  This is the regular (generic) research summary template which is required for all regular applications (unless your protocol fits under the other research summary templates in this category). Use of these instructions is helpful for ensuring that the research summary contains all necessary elements. |  |
| **Standard Research Summary** | |
| **Purpose of the Study** | |
| Objectives & hypotheses to be tested  Using a multidisciplinary and multi-institutional approach, this study aims to yield new insight into the structural and vascular pathophysiology of mild cognitive impairment (MCI), Parkinson’s Disease (PD), Alzheimer’s Disease (AD), frontotemporal dementia (FTD), TBI, concussion, PTSD, and other neurodegenerations as well as Down syndrome which is a risk factor for the development of cognitive impairment and Alzheimer’s disease. We propose to develop and evaluate biomarkers using non-invasive multimodal retinal, choroidal, and optic nerve imaging using optical coherence tomography (OCT) and OCT angiography (OCTA) as well as ultra-widefield color and autofluorescence fundus images to assess the structure and function of the retina and its microvasculature in persons with MCI, PD, AD, FTD, DLB, ALS, HD, MS, TBI, concussion, PTSD, Down syndrome, and other neurodegenerations. We hypothesize that microvascular and structural network alterations in the retina and choroid mirror and possibly precede changes in the cerebral microcirculation seen in MCI and AD and perhaps in other neurodegenerations.  Using advanced image analysis, we aim to evaluate markers of reduced capillary blood flow and non-perfusion in the superficial and deep retinal vascular plexuses and choriocapillaris imaged using OCTA, in a resolution not previously possible, that would complement already established retinal structural markers and increase their sensitivity and specificity in the early detection of some of these conditions. This study looks to provide a proof of concept for OCT, OCTA, and UWF-based retinal structural and microvascular biomarkers as an effective screening tool in cognitive aging. We also aim to quantify progression in OCT and OCTA and UWF metrics over time and correlate with cognitive decline. In order to assess progression and determine the predictive value of these metrics in diagnosing and stratifying neurodegenerative disease and assessing its progression, we would need to reimage the participants who have been previously imaged. In order to do this, we would reconsent subjects who had previously participated in the iMIND study. To that end, all patients, whether previously completed or just beginning, will have images repeated at a return study visit, provided that over time, they are able to physically be able to return for and participate in the study visits and interested in repeat participation and complete testing as was performed in their previous study visit and described above.  These potential biomarkers would complement established retinal structural markers like retinal nerve fiber layer (RNFL) and ganglion cell-inner plexiform layer (GCIPL) thinning in addition to large vessel changes seen on fundus photographs and could increase the sensitivity and specificity of retinal imaging biomarkers. This study will demonstrate the diagnostic potential of retinal structural and microvascular biomarkers and provide a proof of concept for OCT, OCTA, and UWF images as an effective screening tools in cognitive aging. This study may yield new insights into the vascular pathophysiology of AD and other neurodegenerations. We hypothesize that microvascular and structural network alterations in the retina mirror, and may even precede, changes in the cerebral microcirculation and structural changes seen in AD and other neurodegenerations. We will also evaluate the relationship of reduced capillary blood flow and non-perfusion to cognitive function. We will use the images to develop machine-learning models to identify these conditions. |  |
| **Background & Significance** | |
| Should support the scientific aims of the research |  |

| The enormous health and societal costs associated with the dementias and other neurodegenerations are only going to increase as the population grows and ages. The contribution of cerebral small vessel disease to mild cognitive impairment (MCI), Parkinson’s disease (PD), Alzheimer’s disease (AD) and perhaps other neurodegenerations such as frontotemporal dementia (FTD) for example is increasingly recognized. There is also a paucity of literature on microvascular changes in Down syndrome; individuals with Down syndrome have an extra copy of amyloid precursor protein on the extra chromosome 21 and are at higher risk of AD. Postmortem histological studies have demonstrated decreased vessel caliber with collagen deposition, decreased vascular density, and increased cerebral vessel curvature in AD brains. The reduced vascular density may be due to a reduction in angiogenesis caused by the binding of vascular endothelial growth factor to amyloid-β and sequestration in plaques. Cerebral blood flow may be reduced due to these changes.  There is a growing body of evidence suggesting that vascular disease mechanisms play an important role in the risk for age-related cognitive decline and many neurodegenerative disorders. Such small vessel cerebrovascular changes to arterioles, capillaries, and venules are most strongly associated with cognitive impairment; however, they are inaccessible to existing *in vivo* imaging technologies. For example, parenchymal lesions seen on neuroimaging serve as surrogate markers rather than images of the microvascular changes themselves. In contrast, the retinal vascular network can be visualized using photographs and provides a unique “window” to study cerebral microvascular pathology. An association among clinically visible retinal microvascular abnormalities (e.g., retinopathy signs), clinical (e.g., stroke) and subclinical (MRI white matter lesions) cerebral small vessel disease has been described. The retinal and cerebral small blood vessels also share similar embryological origins, anatomical and physiological properties.  Current diagnostic modalities for AD and PD and other dementias and neurodegenerations are limited by cost (MRI, PET), invasiveness (CSF biomarkers), suboptimal specificity and sensitivity (genetic markers, serum amyloid), length of evaluation, and access to specialists including neuropsychological evaluation. Several retinal and choroidal findings associated with AD for example have been detected early in disease course and mirror neurodegenerative changes in the brain. OCT allows quantification of the neurodegeneration of GC-IPL and RNFL thinning, and optic nerve degeneration, which may provide sensitive biomarkers of underlying cerebral processes. OCT has been used to examine the RNFL and GCC in MCI subjects. However, gliosis, which is an early marker of AD pathology, may precede the onset of neuronal death and confound the detection of RNFL thinning. Decreased blood flow in the presence of unchanged RNFL thickness in MCI patients suggests that blood flow abnormalities may precede, rather than follow neurodegeneration in the pathological pathway that results in AD.  Previous studies have focused on clinically visible retinopathy. Using retinal photographs, a sparser and more tortuous retinal microvascular network with narrower retinal arteriolar caliber, wider venular caliber, and smaller arteriolar fractal dimension have been shown in stroke and AD compared with controls. Decreased retinal arteriolar fractal dimension and increased arteriolar tortuosity have been associated with cerebral microbleeds, independent of vascular risk factors and other cerebral markers such as white matter lesions and lacunae, suggesting that these retinal vascular measures may be an early manifestation of cerebral small vessel disease. Compared to controls, retinal venous blood flow in AD is reduced by 39% and in MCI by 19.4%. Generalized arteriolar narrowing and venular dilation have been associated with progression of white matter lesions and lacunar infarcts on MRI. However, visible retinopathy signs are relatively late indicators of target organ damage in the eye and probably reflect advanced stages of structural microvascular damage.  OCTA allows non-invasive, non-dye based *in vivo*, rapid (~5-10 seconds) mapping of the individual superficial and deep retinal capillary network and choriocapillaris with a spatial resolution of ~10μm. OCTA provides visualization of the superficial and deep retinal capillary plexuses at a resolution not previously possible. Quantitative metrics for morphology of retinal capillary density and the flow index in the superficial and deep retinal capillary beds have been recently developed using OCTA to potentially visualize areas of low capillary perfusion that are not yet visible on retinal photographs. It is possible that while early changes in the retinal microvasculature do not reflect the actual severity of cerebral small vessel disease, they may precede its development as well as the development of retinal neurodegeneration.  Collectively, these OCT, OCTA and UWF based retinal and choroidal biomarkers could potentially be more sensitive, specific, and cost effective while serving as an adjunct for neuroimaging, cerebrospinal fluid testing, and genetic testing for the diagnosis of MCI, PD, AD, FTD, and other neurodegenerations such as TBI and PTSD in a unique multidisciplinary approach.  **References** |  |
| --- | --- |

| Benitez-Aguirre P, Craig ME, Sasongko MB, et al. Retinal vascular geometry predicts incident retinopathy in young people with type 1 diabetes: a prospective cohort study from adolescence. *Diabetes Care.* 2011;34 (7):1622-1627.  Fischer VW, Siddiqi A, Yusufaly Y. Altered angioarchitecture in selected areas of brains with Alzheimer's disease. *Acta Neuropathol.* 1990;79(6):672-679.  Tian J, Shi J, Smallman R, Iwatsubo T, Mann DM. Relationships in Alzheimer's disease between the extent of Abeta deposition in cerebral blood vessel walls, as cerebral amyloid angiopathy, and the amount of cerebrovascular smooth muscle cells and collagen. *Neuropathol Appl Neurobiol.* 2006;32(3):332-340.  Dorr A, Sahota B, Chinta LV, et al. Amyloid-beta-dependent compromise of microvascular structure and function in a model of Alzheimer's disease. *Brain.* 2012;135(Pt 10):3039-3050.  Brown WR, Thore CR. Review: cerebral microvascular pathology in ageing and neurodegeneration. *Neuropat hol Appl Neurobiol.* 2011;37(1):56-74.  Hunter JM, Kwan J, Malek-Ahmadi M, et al. Morphological and pathological evolution of the brain microcirculation in aging and Alzheimer's disease. *PLoS One.* 2012;7(5):e36893.  Thal DR, Griffin WS, Braak H. Parenchymal and vascular Abeta-deposition and its effects on the degeneration of neurons and cognition in Alzheimer's disease. *J Cell Mol Med.* 2008;12(5B):1848-1862. Ellis RJ, Olichney JM, Thal LJ, et al. Cerebral amyloid angiopathy in the brains of patients with Alzheimer's disease: the CERAD experience, Part XV. *Neurology.* 1996;46(6):1592-1596.  Jellinger KA. Alzheimer disease and cerebrovascular pathology: an update. *J Neural Transm (Vienna).* 2002; 109(5-6):813-836.  Vinters HV, Wang ZZ, Secor DL. Brain parenchymal and microvascular amyloid in Alzheimer's disease. *Brain Pathol.* 1996;6(2):179-195.  Gorelick PB, Scuteri A, Black SE, et al. Vascular contributions to cognitive impairment and dementia: a statement for healthcare professionals from the american heart association/american stroke association. *St roke.* 2011;42(9):2672-2713.  Kling MA, Trojanowski JQ, Wolk DA, Lee VM, Arnold SE. Vascular disease and dementias: paradigm shifts to drive research in new directions. *Alzheimers Dement.* 2013;9(1):76-92.  Rosenberg GA, Wallin A, Wardlaw JM, et al. Consensus statement for diagnosis of subcortical small vessel disease. *J Cereb Blood Flow Metab.* 2016;36(1):6-25.  Feke GT, Hyman BT, Stern RA, Pasquale LR. Retinal blood flow in mild cognitive impairment and Alzheimer's disease. *Alzheimers Dement (Amst).* 2015;1(2):144-151.  de Jong FJ, Schrijvers EM, Ikram MK, et al. Retinal vascular caliber and risk of dementia: the Rotterdam study. *Neurology.* 2011;76(9):816-821.  Cheung N, Mosley T, Islam A, et al. Retinal microvascular abnormalities and subclinical magnetic resonance imaging brain infarct: a prospective study. *Brain.* 2010;133(Pt 7):1987-1993.  Macgillivray TJ, Patton N, Doubal FN, Graham C, Wardlaw JM. Fractal analysis of the retinal vascular network in fundus images. *Conf Proc IEEE Eng Med Biol Soc.* 2007;2007:6456-6459.  Patton N, Aslam T, Macgillivray T, Pattie A, Deary IJ, Dhillon B. Retinal vascular image analysis as a potential screening tool for cerebrovascular disease: a rationale based on homology between cerebral and retinal microvasculatures. *J Anat.* 2005;206(4):319-348.  Patton N, Pattie A, MacGillivray T, et al. The association between retinal vascular network geometry and cognitive ability in an elderly population. *Invest Ophthalmol Vis Sci.* 2007;48(5):1995-2000.  Wong TY, Klein R, Couper DJ, et al. Retinal microvascular abnormalities and incident stroke: the Atherosclerosis Risk in Communities Study. *Lancet.* 2001;358(9288):1134-1140.  Lesage SR, Mosley TH, Wong TY, et al. Retinal microvascular abnormalities and cognitive decline: the ARIC 14-year follow-up study. *Neurology.* 2009;73(11):862-868.  Baker ML, Marino Larsen EK, Kuller LH, et al. Retinal microvascular signs, cognitive function, and dementia in older persons: the Cardiovascular Health Study. *Stroke.* 2007;38(7):2041-2047.  Qiu C, Cotch MF, Sigurdsson S, et al. Cerebral microbleeds, retinopathy, and dementia: the AGES-Reykjavik Study. *Neurology.* 2010;75(24):2221-2228.  Liew G, Wang JJ, Cheung N, et al. The retinal vasculature as a fractal: methodology, reliability, and relationship to blood pressure. *Ophthalmology.* 2008;115(11):1951-1956.  Haan M, Espeland MA, Klein BE, et al. Cognitive function and retinal and ischemic brain changes: the Women's Health Initiative. *Neurology.* 2012;78(13):942-949.  Cheung N, Liew G, Lindley RI, et al. Retinal fractals and acute lacunar stroke. *Ann Neurol.* 2010;68(1):107-111.  Wong TY, Klein R, Klein BE, Tielsch JM, Hubbard L, Nieto FJ. Retinal microvascular abnormalities and their relationship with hypertension, cardiovascular disease, and mortality. *Surv Ophthalmol.* 2001;46(1):59-80. Hinton DR, Sadun AA, Blanks JC, Miller CA. Optic-nerve degeneration in Alzheimer's disease. *N Engl J Med.* 1986;315(8):485-487.  Ascaso FJ, Cruz N, Modrego PJ, et al. Retinal alterations in mild cognitive impairment and Alzheimer's disease: an optical coherence tomography study. *J Neurol.* 2014;261(8):1522-1530.  Cheung CY, Ong YT, Hilal S, et al. Retinal ganglion cell analysis using high-definition optical coherence tomography in patients with mild cognitive impairment and Alzheimer's disease. *J Alzheimers Dis.* 2015;45 (1):45-56.  Kesler A, Vakhapova V, Korczyn AD, Naftaliev E, Neudorfer M. Retinal thickness in patients with mild cognitive impairment and Alzheimer's disease. *Clin Neurol Neurosurg.* 2011;113(7):523-526.  Liu D, Zhang L, Li Z, et al. Thinner changes of the retinal nerve fiber layer in patients with mild cognitive impairment and Alzheimer's disease. *BMC Neurol.* 2015;15:14. |  |
| --- | --- |

| Oktem EO, Derle E, Kibaroglu S, Oktem C, Akkoyun I, Can U. The relationship between the degree of cognitive impairment and retinal nerve fiber layer thickness. *Neurol Sci.* 2015;36(7):1141-1146.  Paquet C, Boissonnot M, Roger F, Dighiero P, Gil R, Hugon J. Abnormal retinal thickness in patients with mild cognitive impairment and Alzheimer's disease. *Neurosci Lett.* 2007;420(2):97-99.  Shen Y, Liu L, Cheng Y, et al. Retinal nerve fiber layer thickness is associated with episodic memory deficit in mild cognitive impairment patients. *Curr Alzheimer Res.* 2014;11(3):259-266.  Shi Z, Wu Y, Wang M, et al. Greater attenuation of retinal nerve fiber layer thickness in Alzheimer's disease patients. *J Alzheimers Dis.* 2014;40(2):277-283.  Knoll B, Simonett J, Volpe NJ, et al. Retinal nerve fiber layer thickness in amnestic mild cognitive impairment: Case-control study and meta-analysis. *Alzheimers Dement (Amst).* 2016;4:85-93.  Ingelsson M, Fukumoto H, Newell KL, et al. Early Abeta accumulation and progressive synaptic loss, gliosis, and tangle formation in AD brain. *Neurology.* 2004;62(6):925-931.  Mandybur TI. Cerebral amyloid angiopathy and astrocytic gliosis in Alzheimer's disease. *Acta Neuropathol.* 1 989;78(3):329-331.  Bates KA, Fonte J, Robertson TA, Martins RN, Harvey AR. Chronic gliosis triggers Alzheimer's disease-like processing of amyloid precursor protein. *Neuroscience.* 2002;113(4):785-796.  Cheung CY, Ong YT, Ikram MK, et al. Microvascular network alterations in the retina of patients with Alzheimer's disease. *Alzheimers Dement.* 2014;10(2):135-142.  Frost S, Kanagasingam Y, Sohrabi H, et al. Retinal vascular biomarkers for early detection and monitoring of Alzheimer's disease. *Transl Psychiatry.* 2013;3:e233.  Ong YT, De Silva DA, Cheung CY, et al. Microvascular structure and network in the retina of patients with ischemic stroke. *Stroke.* 2013;44(8):2121-2127.  Hilal S, Ong YT, Cheung CY, et al. Microvascular network alterations in retina of subjects with cerebral small vessel disease. *Neurosci Lett.* 2014;577:95-100.  Doubal FN, MacGillivray TJ, Patton N, Dhillon B, Dennis MS, Wardlaw JM. Fractal analysis of retinal vessels suggests that a distinct vasculopathy causes lacunar stroke. *Neurology.* 2010;74(14):1102-1107.  Sherman TF. On connecting large vessels to small. The meaning of Murray's law. *J Gen Physiol.* 1981;78 (4):431-453.  Hubbard LD, Brothers RJ, King WN, et al. Methods for evaluation of retinal microvascular abnormalities associated with hypertension/sclerosis in the Atherosclerosis Risk in Communities Study. *Ophthalmology.* 1 999;106(12):2269-2280.  Sun C, Wang JJ, Mackey DA, Wong TY. Retinal vascular caliber: systemic, environmental, and genetic associations. *Surv Ophthalmol.* 2009;54(1):74-95.  Schreiber S, Bueche CZ, Garz C, et al. The pathologic cascade of cerebrovascular lesions in SHRSP: is erythrocyte accumulation an early phase? *J Cereb Blood Flow Metab.* 2012;32(2):278-290.  Berisha F, Feke GT, Trempe CL, McMeel JW, Schepens CL. Retinal abnormalities in early Alzheimer's disease. *Invest Ophthalmol Vis Sci.* 2007;48(5):2285-2289.  Ikram MK, de Jong FJ, Vernooij MW, et al. Retinal vascular calibers associate differentially with cerebral gray matter and white matter atrophy. *Alzheimer Dis Assoc Disord.* 2013;27(4):351-355.  Ikram MK, Ong YT, Cheung CY, Wong TY. Retinal vascular caliber measurements: clinical significance, current knowledge and future perspectives. *Ophthalmologica.* 2013;229(3):125-136.  Classification of diabetic retinopathy from fluorescein angiograms. ETDRS report number 11. Early Treatment Diabetic Retinopathy Study Research Group. *Ophthalmology.* 1991;98(5 Suppl):807-822.  Jia Y, Morrison JC, Tokayer J, et al. Quantitative OCT angiography of optic nerve head blood flow. *Biomed Opt Express.* 2012;3(12):3127-3137.  Agemy SA, Scripsema NK, Shah CM, et al. Retinal Vascular Perfusion Density Mapping Using Optical Coherence Tomography Angiography in Normals and Diabetic Retinopathy Patients. *Retina.* 2015;35(11): 2353-2363.  Hwang TS, Gao SS, Liu L, et al. Automated Quantification of Capillary Nonperfusion Using Optical Coherence Tomography Angiography in Diabetic Retinopathy. *JAMA Ophthalmol.* 2016;134(4):367-373. Gharbiya M, Trebbastoni A, Parisi F, et al. Choroidal thinning as a new finding in Alzheimer's disease: evidence from enhanced depth imaging spectral domain optical coherence tomography. *J Alzheimers Dis.* 2 014;40(4):907-917.  Cheung CY, Hsu W, Lee ML, et al. A new method to measure peripheral retinal vascular caliber over an extended area. *Microcirculation.* 2010;17(7):495-503. |  |
| --- | --- |
| **Design & Procedures** | |
| Describe the study, providing details regarding the study intervention (drug, device, physical procedures, manipulation of the subject or the subject’s environment, etc.). Discuss justifications for placebo control, discontinuation or delay of standard therapies, and washout periods if applicable. Identify procedures, tests and interventions performed exclusively for research purposes or more frequently than standard of care. Include alternative therapies, concurrent therapies discontinued per protocol, risk benefit ratio, and use of tissue/specimens. Discuss monitoring during washout periods if applicable. Include brief description of follow-up, if any. |  |

| Design: Cross-sectional and longitudinal case-control study of patients with MCI, PD, AD, DLB, FTD, multiple sclerosis, ALS, Huntington’s, and other neurodegenerations such as TBI, concussion, and PTSD, among others and age-race-sex matched controls, for at least 2000 participants from Duke Health.  Controls will be recruited from the relatives/caregivers of the patients, Duke Alzheimer’s Disease Research Center (ADRC), Duke Aging Center, or will be patients themselves, and will not have a diagnosis of MCI, PD, AD, FTD, TBI, concussion, PTSD, or other neurodegenerative conditions. Participants who are interested and available to return for follow-up visits will be reconsented and imaged per protocol up to 4 times within a year. This will allow a subset to have longitudinal follow-up participation. Retrospective data  /images that were captured from Pro00082598 will be included in this study as well for the retrospective portion. This will contain up to 2000 previously imaged subjects for this initial study.  Protocol:  Information such as age, sex, years of education, and some health history questions will be collected. Corrected visual acuity will be measured in both eyes. A Mini-Mental State Examination (MMSE) will also be conducted. Those with Down syndrome will have had IQ testing in the Duke genetics clinic and thus will not complete the MMSE.  All study images will be captured by iMIND study key personnel who will have been trained. No eye drops will be used. No eye contact will occur. No x-rays are used.  Optical Coherence Tomography (OCT) images will be obtained using the Zeiss Cirrus HD-5000 with AngioPlex machine: OCT macula cube, OCT macula HD-21 scan, and OCT optic disc cube. Parameters collected include central subfield thickness (CST), subfoveal choroidal thickness, retinal nerve fiber layer (RNFL) thickness, ganglion cell-inner plexiform layer (GCIPL) thickness. The choroid will be further measured using COIN software platform created by collaborators Tan Tock Seng Hospital in Singapore to assess choroidal vascularity index by calculating total choroidal area and luminal area. Choroidal thinning may be related to a series of pathologic events (e.g. inflammatory cascade) triggered by amyloid-β deposition in the brain and is associated with AD.  OCT Angiography (OCTA) images will also be obtained on the same Zeiss machine: A 3×3 mm and 6x6 mm scanning area centered on the fovea and a 4.5 x 4.5 mm area centered on the optic disc will be captured. Optic disc scans will be captured using the OCTA Scan 4.5x4.5 mm for imaging optic disc (Cirrus software version 11.0). This in particular is an investigational software and is not approved by the U.S. Food and Drug Administration (FDA). This software has been added to the US FDA-approved Zeiss Cirrus HD-5000 OCT with AngioPlex OCTA device to provide the capability of taking OCT and OCTA images of the optic nerve. This will allow for quantification of the Radial Peripapillary Capillaries (RPC) metrics: Capillary Perfusion Density (CPD) and Capillary Flux Index (CFI). Three layers from the en face OCTA will be analyzed: superficial retinal capillary plexus, deep retinal capillary plexus, and choriocapillaris. The superficial plexus consists of the capillaries between the inner limiting membrane (ILM) and the inner plexiform layer (IPL). The deep plexus consists of the capillaries between the IPL and the outer plexiform layer (OPL) (including inner nuclear layer). The choriocapillaris consists of capillaries in a 30-µ m thick layer posterior to the retinal pigment epithelium- Bruch membrane junction. OCTA images will be converted from a gray scale to black and white using the vessel skeletonization technique. Parameters measured using OCTA in the superficial and deep capillary plexuses will include the foveal avascular zone (FAZ), vessel density, and perfusion density. In quantifying perfusion density for the deeper vascular layers, vessel density information from more superficial segments is subtracted to allow for accurate analysis of each layer individually. This analysis will be performed for all 3 layers in both the 3×3-mm scans centered on the fovea and the 4.5 x 4.5 mm scans centered on the optic nerve for each subject.  **Ultra-widefield (UWF) color and autofluorescence fundus photographs** will be obtained on the Optos California camera. Undilated ultra-widefield fundus and autofluorescence photographs will be analyzed using the VAMPIRE software in all study subject groups[**(http://vampire.computing.dundee.**](http://vampire.computing.dundee/) **ac.uk/publications.html)** for the already established parameters of retinal vessel caliber, retinal branching angles and tortuosity and retinal fractal dimension. De-identified fundus photographs, stripped of all patient identifiers and patient data will be transferred to the University of Edinburgh in Scotland, UK for analysis using the VAMPIRE software as well as to Queens University in Belfast, Ireland, UK for additional analyses via Duke Box. Data transfer agreement is in place.  De-identified images will be used for the development of machine learning algorithms to differentiate the various neurodegenerations from each other and from  controls. These algorithms are for exploratory purposes only and will  not be used to assess or treat subjects in this study. We may opt to share the de-  identified images with the US Food and Drug Administration (FDA) for approval of a diagnostic index to diagnose AD or any of these neurodegenerations.  For the purpose of review prior to publication, de-identified images will also be shared with the following institutions:  TAN TOCK SENG HOSPITAL PTE LTD - Prof Rupesh Agrawal with DTA in place |  |
| --- | --- |

| The Queen's University of Belfast - Imre Lengyel, M.D. with DTA in place.  De-identified images will be shared using password-protected Duke Box [(https://box.duke.edu/).](https://box.duke.edu/))  **Inclusion and Exclusion Criteria:**  We will enroll patients with neurodegenerative disease (MCI, PD, AD, multiple sclerosis, ALS, Huntington’s disease, frontotemporal dementia, DLB, and other neurodegenerations such as TBI, concussion, and PTSD, Down syndrome) being evaluated at Duke Health, including in the Duke Memory Disorders clinics and other Duke neurology clinics on Morreene Road or the Duke Genetics Clinics as well as age-gender-race-matched controls which could also include healthy patients from these clinics. Participants will be 18 years or older at the time of enrollment. Dr Grewal and Dr Fekrat and other iMIND staff members will prescreen clinic schedules through their secure Epic login to help identify patients eligible for the study. Potentially eligible patients may be contacted using IRB approved script via Duke iMIND email and/or telephone to inform them about the iMIND study in advance of their Duke clinic appointment.  Exclusion criteria include the inability to cooperate with or complete testing or other neurologic or age-related ocular conditions that would impact image acquisition. Eyes that have had intraocular surgery, other than cataract surgery, will be excluded. If two eyes satisfy the inclusion criteria, both eyes will be included in the study. If one eye satisfies the inclusion criteria, the eye that qualifies will be included in the study.  **Research Activities:**  Ophthalmologic examination and Retinal Imaging Protocol: All participants will undergo assessment of corrected distance visual acuity. A Mini-Mental State Examination (MMSE) will also be conducted as well as a pen-light exam of the front of the eye. Subjects with Down Syndrome who have had IQ testing by their clinician will not undergo MMSE testing. Data collection will include age, sex, years of education and other health history questions. Participants will also undergo OCT and OCTA using the Zeiss Cirrus machine (Cirrus AngioPlex, Carl Zeiss Meditec, Dublin, CA) and fundus images using the Optos California fundus camera (Optos, Marlborough, MA, USA). The devices used for retinal and choroidal imaging are FDA-approved and utilized in everyday clinical practice. Multimodal imaging testing takes approximately 30 minutes or so.  Study participation may involve additional visits as needed as determined by the quality of the images obtained. Participants who are interested and available to return for follow-up visits, up to 9 times within a year, will be reconsented and imaged per protocol. This will allow a subset to have longitudinal follow-up participation.  Participants can choose to stop participating at any time without consequence or penalty. If the participant is a Duke employee or student, the job status and/or grades will not be affected.  De-identified images and associated quantitative metrics will be analyzed in Duke Compute Cluster (DCC). |  |
| --- | --- |
| **Selection of Subjects** | |
| List inclusion/exclusion criteria and how subjects will be identified.  **Inclusion and Exclusion Criteria**  We will enroll patients with neurodegenerative disease (MCI, PD, AD, FTD, DLB, ALS, MS, HD, TBI, concussion, PTSD and other neurodegenerations as well as Down Syndrome) being evaluated at Duke Health and in the Duke Memory Disorders clinics and other Duke neurology clinics at Duke Morreene Road or the Duke Genetics Clinics and community referrals as well as age-gender-race-matched controls which could also include healthy patients from Duke Health. Participants will be 18 years or older at the time of enrollment. Dr Grewal and Dr Fekrat or other iMIND staff members will prescreen clinic schedules through their secure Epic login to help identify patients potentially eligible for the study. Potentially eligible patients may be contacted using IRB-approved script via Duke iMIND email and/or telephone to inform them about the iMIND study in advance of their Duke clinic appointment.  Exclusion criteria include the inability to cooperate with or complete testing or other neurologic or age-related ocular conditions that would impact image acquisition. Eyes that have had intraocular surgery, other than cataract surgery, will be excluded. If two eyes satisfy the inclusion criteria, both eyes will be included in the study. If one eye satisfies the inclusion criteria, the eye that qualifies will be included in the study. |  |

| Participants previously enrolled in Pro00082598 will have their data/images included in this study in the prospective portion. |  |
| --- | --- |
| **Subject Recruitment and Compensation** | |
| Describe recruitment procedures, including what method(s) will be used, when the study will be introduced to potential participants and by whom. If any follow-up contact is planned, describe the proposed method and timing. Describe how you will ensure that subject selection is equitable and all relevant demographic groups have access to study participation (per 45 CFR 46.111(a) (3)). Include information about how many DUHS participants will be recruited. If participants are to be compensated and/or reimbursed, provide specific prorated amounts to be provided for expenses such as travel and  /or lost wages, and/or for inducement to participate.  Subjects will be recruited from Duke Health including from among the patients of the Duke Memory Disorders Clinics, Duke Neurology Clinics, Duke Alzheimer’s Disease Research Center (ADRC), Duke Aging Center, the Duke Genetics Clinics or any other Duke referral or community referrals through community engagement. Also, from referrals from the Duke Dementia Family Support program and the NC Registry for Brain Health or other community sources. Duke employees or students are permitted to participate.  Duke Health or community individuals who do not have a known neurodegenerative diagnosis such as MCI, PD, AD, TBI, concussion, Down, PTSD or other neurodegeneration will serve as controls or normal subjects. The target enrollment will be up to 4000 subjects.  DEDUCE search query in PACE will be conducted & used for recruitment as well. Potential participants may be contacted in advance of their Duke appointment, via IRB-approved phone script, email template, or US postal mail language following protocol. Patients who have opted out of research contact, showing Declined Status in Maestro, will not be contacted. Patients will not be contacted more than three times, including via phone, email, and face-to-face interaction. If a patient states they would like to opt out, the study team will follow the Duke Recruitment and Engagement policy. On the day before scheduled clinic patients are scheduled to come in for their Duke appointment, the iMIND study team will notify via send secure email their physician or provider about potentially eligible study patients who meet eligibility criteria and have an Interested Status in Maestro &/or have expressed interest in the iMIND study via email or phone call prior to their scheduled appointment. In the clinics, Duke neurologists and providers will mention the study and will ask their patient’s permission to be contacted by a member of the iMIND team. They may also share a study flyer or Smartphrase in the after-visit summary. If the patient indicates they are interested, the study staff team will escort the patient to the iMIND study room to discuss the study further and answer questions.  We also aim to quantify progression in OCT, OCTA, and UWF imaging metrics over time and their correlation with  cognitive decline. In order to assess progression and determine the predictive value of these metrics in diagnosing neurodegenerative disease and assessing its progression,  we will call back participants who previously participated in this iMIND study to gauge interest in another study visit. To that end, all patients, whether previously participated or not, will participate  in the same study protocol.  Subjects will be responsible for all costs related to their medical care. There will be no charges to the subject related to the study. Compensation will be provided to the subjects with known disease as well the controls. All subjects will be reimbursed $20 with a check from Duke for time, travel, and parking per imaging session approximately up to a total of potentially $200. |  |
| **Consent Process** | |
| Complete the consent section in the iRIS Submission Form. |  |
| **Subject’s Capacity to Give Legally Effective Consent** | |
|  |  |

| If subjects who do not have the capacity to give legally effective consent are included, describe how diminished capacity will be assessed. Will a periodic reassessment occur? If so, when? Will the subject be consented if the decisional capacity improves?  Dr. Andy Liu or other Duke neurologists and providers and Dr. Kim Johnson or other physicians or non-MD providers in the Morreene Road clinics will evaluate a subject’s capacity to provide consent. If the participant is deemed unable to provide informed consent (ie lacks the capacity to make an informed decision regarding participation) as based on the Capacity Assessment, a legally authorized representative (LAR) will review the informed consent with study staff and sign on the subject’s behalf. |  |
| --- | --- |
| **Study Interventions** | |
| If not already presented in the Design & Procedures section, describe study-related treatment or use of an investigational drug or biologic (with dosages), or device, or use of another form of intervention (i. e., either physical procedures or manipulation of the subject or the subject’s environment) for research purposes.  The study consists of the measurement of corrected distance visual acuity in both eyes of each study participant, the administration of the Mini-Mental State Examination (MMSE), and the acquisition of undilated multimodal retinal and choroidal imaging of both  eyes using the Zeiss Cirrus HD-5000 with AngioPlex and Optos California imaging machines. No eye drops will be used. There will be no contact with the eyes themselves. There are no x-rays. Study staff may need to review study participants’  neurocognitive medical records. |  |
| **Risk/Benefit Assessment** | |
| Include a thorough description of how risks and discomforts will be minimized (per 45 CFR 46.111(a) (1 and 2)). Consider physical, psychological, legal, economic and social risks as applicable. If vulnerable populations are to be included (such as children, pregnant individuals, imprisoned persons or cognitively impaired adults), what special precautions will be used to minimize risks to these subjects? Also identify what available alternatives the person has if he/she chooses not to participate in the study. Describe the possible benefits to the subject. What is the importance of the knowledge expected to result from the research?  The risks of the distance visual acuity testing and the retinal and choroidal imaging are very low. Minor fatigue from testing is  possible; breaks will be introduced as needed during the testing. No eye drops will be employed. There is no physical risk to the administration of the MMSE testing. For many of the case participants, they will likely be already be familiar with MMSE testing. For controls with normal cognition, the MMSE testing will be normal.  Minor discomfort from the imaging camera lights during imaging of both eyes is a possible but unlikely outcome. No eye drops will be used. There will be no contact with the eyes themselves. There are no x-rays. The minor risks are justified by the potential of this clinical research to be translatable and thus contribute to potentially better and easier diagnosis and risk stratification of neurodegenerative conditions, including but not limited to mild cognitive impairment (MCI), Parkinson's Disease (PD), Alzheimer’s Disease (AD), and other neurodegenerations such as frontotemporal dementia (FTD), dementia of Lewy bodies, multiple sclerosis, amyotrophic lateral sclerosis (ALS), Huntington’s disease, Down syndrome, traumatic brain injury (TBI), posttraumatic stress disorder (PTSD), concussion, and normal cognition, in the future.  Subjects will directly benefit from the study by receiving visual acuity testing and OCT, OCTA, and UWF imaging and all will be at no charge. These images serve as a screening tool for retinal diseases. The main findings from the images can be shared with the subject and referral will be made by one of the PI’s to an appropriate ophthalmologist or subspecialist if needed. The study will not provide refractive prescriptions to the participants. |  |

| Cognitively impaired individuals will be assessed by the referring neurologist or neurology provider and/or iMIND study staff using the Capacity Assessment form. If the individual is not able to provide informed consent, a legally authorized representative (LAR) will provide informed consent. The LAR or caregiver will accompany the individual throughout the study visit.  De-identified images and associated quantitative data will be analyzed in Duke Compute Cluster (DCC). Should an individual choose not to participate, it will not affect their medical care at Duke Health. |  |
| --- | --- |
| **Costs to the Subject** | |
| Describe and justify any costs that the subject will incur as a result of participation; ordinarily, subjects should not be expected to pay for research without receiving direct benefit.  Subjects will be responsible for all costs related to their medical care. There will be no charges to the subject related to the study. |  |
| **Data Management, Analysis and Statistical Considerations** | |
| Describe endpoints and power calculations. Provide a detailed description of how study data will be analyzed, including statistical methods used, and how ineligible subjects will be handled and which subjects will be included for analysis. Include planned sample size justification. Provide estimated time to target accrual and accrual rate. Describe interim analysis including plans to stop accrual during monitoring. Phase I studies, include dose escalation schema and criteria for dose escalation with definition of MTD and DLT.  **For external collaborators/personnel** who are NOT ENGAGED in the research but are handling de-identified or a limited data set of **Duke** data, include their name, role and a description of the data.  At least 3000 participants will be recruited. A departmental statistician will be on this IRB and part of our study to assist with statistical analyses. ANOVA and generalized estimating equations will be used to determine the significance of differences in retinal microvasculature and structure between and among the study groups. Linear regression will be used to assess if the relationship between retinal and choroidal imaging biomarkers and neurodegenerative condition severity by group is significantly different from 0. An analysis of co-variance with term for interaction of group and density will be employed to determine whether the relationship between biomarkers and neurodegenerative condition severity is different between groups. Statistical analyses will be performed in SAS 9.2 (SAS Institute Inc., Cary, NC, USA).  The large number of subjects is needed for several reasons   1. Larger number of participants is needed for machine learning model training, validation, and testing 2. iMIND clinical research cohorts need to be diverse and inclusive, 3. There are at least 10 neurodegenerative diagnoses as well as controls with normal cognition that will be included in this study.   De-identified images and quantitative data will be analyzed in Duke Compute Cluster (DCC).  The data consists of approximately 3000 Enhanced Depth Imaging Optical Coherence Tomography (EDI OCT) stored in the Zeiss Cirrus Viewer Software in the iMIND lab on a password protected Duke desktop computer. These images, belonging to approximately 1000 unique participants, will be exported from the software onto the local computer's disk by the Discloser. These image files will then be de-identified and re-titled by the Discloser before being shared with the Recipient. The Recipient will first manually analyze all de-identified image files. Then, these same de-identified images files as well as manual analysis results will be transferred to the Duke Compute Cluster (DCC) via Globus for the purposes of training a Machine Learning model capable of automatically analyzing the images. Once training and evaluation of the  Machine Learning model is complete, all source data will be deleted from the Duke Compute Cluster.  Duke undergraduate student Yana Shah is a volunteer who will work on deidentified image analysis of OCT and OCTA data. |  |
| **Data & Safety Monitoring** | |
|  |  |

| Summarize safety concerns, and describe the methods to monitor research subjects and their data to ensure their safety, including who will monitor the data, and the frequency of such monitoring. If a data monitoring committee will be used, describe its operation, including stopping rules and frequency of review, and if it is independent of the sponsor (per 45 CFR 46.111(a) (6)).  Data collection will be monitored by the study staff on a regular basis. No long-term safety concerns are anticipated as a result of study interventions. Although unlikely, short-term discomfort or side effects from the retinal and choroidal imaging testing will be monitored by study staff and reported to appropriate oversight groups at Duke Health. These events will be monitored by Dr. Grewal, Dr. Fekrat, and the study staff. After-hours contact information will be provided to all study participants in case of adverse events, although unlikely. |  |
| --- | --- |
| **Privacy, Data Storage & Confidentiality** | |
| Complete the Privacy and Confidentiality section of the iRIS submission form. |  |
| **Study Scope** | |
| **Does this study have a cancer focus? Cancer focus includes studies that enroll >50% oncology or malignant hematology patients; or, preventing, detecting, and diagnosing cancer or understanding the impact of cancer on patients and their caretakers.** | |
|  Yes  No |  |
| **Does this study involve the use of a drug, biologic, food, or dietary supplement?** | |
|  Yes  No |  |
| **Does this study involve the use of a medical device, an algorithm (whether computer based or not), an in vitro diagnostic test, or samples to look for biomarkers?** | |
|  Yes  No |  |
| **Does this study involve a Humanitarian Use Device (HUD)?** | |
|  Yes  No |  |
| **Does this study employ magnetic resonance, including imaging (MRI), spectroscopy (MRS), angiography (MRA) or elastography (MRE) beyond the standard of care?** | |
|  Yes  No |  |
| **Does this study specify or require the performance of diagnostic procedures using ionizing radiation (x-rays, DEXA, CT scans, nuclear medicine scans, etc.) that are beyond the standard of care?** | |
|  Yes  No |  |
| **Does this study specify or require the performance of therapeutic procedures using ionizing radiation (accelerator, brachytherapy or systemic radionuclide therapy) that are beyond the standard of care?** | |
|  |  |

|  Yes  No |  |
| --- | --- |
| **Does this study specify or require the use of a laser system for diagnosis or therapy that is beyond the standard of care (excludes the use of lasers as a standard surgical instrument)?** | |
|  Yes  No |  |
| **Will the participant be subjected to increased or decreased ambient pressure?** | |
|  Yes  No |  |
| **Do you plan to recruit participants from or conduct study activities at Duke Regional?** | |
|  Yes  No |  |
| **Do you plan to recruit participants from or conduct study activities at Duke Raleigh?** | |
|  Yes  No |  |
| **Does this study include using the Duke logo in any advertisements?** | |
|  Yes  No |  |
| **Is this study retrospective, prospective, or both?** | |
| "Retrospective" means that data or samples already in existence (collected prior to the study submission) will be used.  "Prospective" means there will be data or samples collected in this study for research purposes.  Retrospective Prospective  Retrospective and Prospective  If the study is both retrospective and prospective: Is this a review solely of information collected for non-research purposes (i.e. a review of medical records)?   Yes  No |  |
| **Does this protocol include any research using botulinum toxin, including the FDA-approved clinical product (Botox)?** | |
|  Yes  No |  |
| **Does this protocol involve the administration of any of the following materials to humans?** | |
|  |  |

| **Category of Investigational Product** | **Examples** |
| --- | --- |
| Any mRNA | Pfizer or Moderna COVID-19 vaccines |
| Any viral vector | AAV vector, adenoviral vector (e.g., J&J or Astrazeneca COVID-19 vaccine) |
|  | |

| Any genetically-modified cells | CAR-T cells (e.g., Kymriah, or other autologous cells) |
| --- | --- |
| Any genetically-modified organisms (virus, bacterium, or other agents) | Oncolytic viruses (Imlygic, others), certain live attenuated vaccines, challenge viruses or challenge bacteria |
| Any plasmid DNA | DNA vaccines |
| Any other recombinant or synthetic nucleic acid (DNA, RNA, others) |  |

|  Yes  No |  |
| --- | --- |
| **Subject Population Groups and Enrollment** | |
| **Population Groups (Select targeted population groups only):** | |
| Adults  Minors who are Wards of State Minors  Duke Patients Pregnant individuals Fetuses  Imprisoned Persons  Adults incapable of giving consent Adults with diminished capacity Disabled subjects  Students Employees Healthy Controls Deceased subjects Blanket Protocol  Students and employees over whom Key Personnel have a supervisory role may not be enrolled in this study. |  |
| **Please select any population groups excluded from participation in this study:** | |
| Pregnant individuals |  |
| **Maximum number of subjects to be consented at Duke:** | |
| Enter a single number. If you anticipate consenting a range of subjects, enter the **upper** limit of the range. The number should represent the maximum number of subjects for the life of the study.  3000 |  |
| **Maximum number of subjects to be consented at all sites:** | |
|  |  |

| Enter a single number. If you anticipate consenting a range of subjects, enter the **upper** limit of the range. The number should represent the maximum number of subjects for the life of the study.  3000 |  |
| --- | --- |
| **Maximum number of patient records / samples to be used:** | |
| 3000 |  |
| **Subject Procedures and Costs** | |
| **Biobank - Does this study involve the collection, use, tracking, banking (storage) or distribution of human biological specimens?** | |
| Human biological specimens include blood or its components, healthy or diseased tissue, bodily fluids, DNA  /RNA or human stem cells.  Yes No |  |
| **Procedures** | |
| **Check all that apply:**  Genetic Testing Gene Transfer DNA Banking  Testing for Reportable Infectious Diseases Human Cell Banking  *Use of Human Embryonic Stem Cells  *Use of Human-induced Pluripotent Stem Cells  *Use of Other Cells Derived from Human Embryos  *Use of Human/Animal Chimeric Cells  *Specialized Cell Populations for Cell Therapy Use of Human Tissue  Use of Bodily Fluids  Use of Blood (or its components) Not Applicable |  |
| **Will blood be drawn in this study for research purposes?** | |
| Yes No |  |
| **Will the Operating Room be used in this study?** | |
| Include only research time, not clinical care time.  Yes No |  |
| **Will there be extra costs to subjects or insurance as a result of the research (e.g. tests, hospitalization)?** | |
| Yes No |  |
| **Will there be Subject Compensation?** | |
|  |  |

Yes	No

## Compensation for Travel / Lost Income (in USD):

20

## Other Subject Compensation:

Subject compensation per imaging/re-imaging up to a possible $200.

# Subject Recruitment Materials

**For each document to be reviewed, use the table below to provide the following information:**

## Attach a copy of each advertisement to be used with this study in the Initial Submission Packet. If any advertisement will have multiple wording variations, attach a copy of each version of the advertisement. For media materials (i.e., videos, radio ads, commercials), include transcripts and links.

If an advertisement includes the Duke logo, be sure to indicate “yes” in Study Scope section. The IRB must approve all study advertisements used to recruit.

Types of subject recruitment materials include, but are not limited to, the following:

**Direct Advertising**

Posters Billboards Flyers Brochures

**Media Advertising** Newspaper Ads Magazine Ads

Radio Ads

TV commericals / Video Internet website

Social Media

**Other Types of Advertising**

Newsletter Email

Postcards / Letters

(Note: Doctor-to-Doctor letters do not require IRB approval)

| **Document name** | **Material category** | **Location material displayed** | **Has this material previously been**  **approved by the IRB?** |
| --- | --- | --- | --- |
|  | Billboard / Flyer / Poster  Brochure Internet website  / Email | Please be specific. For example, "Duke" would not be an appropriate location. "Duke Hospital Television" would be an appropriate response. |  |

|  | iMInd Controls study Flyer |  | Letter / Postcard  Phonescript |  | Duke Health, Duke  Memory Disorders Clinics and Neurology Clinics at Moreene Rd., Duke Dementia Family Support Program and the NC Registry for Brain Health as well as throughout the community.  Included as a Smartpharse in after visit summary in Epic |  | Yes | No |
| --- | --- | --- | --- | --- | --- | --- | --- | --- |
|  | | | Radio |  |  |  |  |  |
|  |  |  | Television / Video |  |  |  |  |  |
|  |  |  | Newsletter / Newspaper / Magazine |  |  |  |  |  |
|  |  |  | Other |  |  |  |  |  |
|  |  |  |  |  | | |  |  |
|  | | | Billboard / Flyer / Poster | Please be specific. For example, "Duke" would not be an appropriate location. "Duke Hospital Television" would be an appropriate response.  Duke Health, Duke Memory Disorders Clinics and Neurology Clinics at Moreene Rd., Duke Dementia Family Support Program and the NC Registry for Brain Health as well as throughout the community.  Included as a Smartpharse in after visit summary in Epic | | |  |  |
|  | | | Brochure |  |  |  |  |  |
|  | | | Internet website  / Email |  |  |  |  |  |
| iMIND Case Study Flyer | | | Letter / Postcard Phonescript  Radio |  |  |  | Yes | No |
|  | | | Television / Video |  |  |  |  |  |
|  | | | Newsletter / Newspaper / Magazine |  |  |  |  |  |
|  | | | Other |  |  |  |  |  |
|  | | | Billboard / Flyer / Poster | Please be specific. For example, "Duke" would not be an appropriate location. "Duke Hospital Television" would be an appropriate response.  Will be emailed to eligible subjects under the cold call policy | | |  |  |
|  | | | Brochure |  |  |  |  |  |
|  | | | Internet website  / Email |  |  |  |  |  |
| iMIND email language | | | Letter / Postcard Phonescript  Radio |  |  |  | Yes | No |
|  | | | Television / Video |  |  |  |  |  |
|  | | | Newsletter / Newspaper / Magazine |  |  |  |  |  |
|  | | | Other |  |  |  |  |  |
|  | | | Billboard / Flyer / Poster | Please be specific. For example, "Duke" would not be an appropriate location. "Duke Hospital Television" would be an appropriate response. | | |  |  |
|  | | | Brochure |  |  |  |  |  |
|  | | | Internet website  / Email |  |  |  |  |  |
|  | | | Letter / Postcard |  |  |  |  |  |
| iMIND phone script | | | Phonescript Radio |  |  |  | Yes | No |

|  | Television / Video  Newsletter / Newspaper / Magazine  Other | Will be used to explain the study to participants who respond to email or letter |  | |
| --- | --- | --- | --- | --- |
|  | Billboard / Flyer / Poster | Please be specific. For example, "Duke" would not be an appropriate location. "Duke Hospital Television" would be an appropriate response.  Will be mailed to eligible subjects under the cold call policy |  |  |
|  | Brochure |  |  |  |
|  | Internet website  / Email |  |  |  |
| iMIND US postal letter | Letter / Postcard Phonescript  Radio |  | Yes | No |
|  | Television / Video |  |  |  |
|  | Newsletter / Newspaper / Magazine |  |  |  |
|  | Other |  |  |  |

| **Consent Process** | |
| --- | --- |
| **Attach draft consent forms in the Initial Review Submission Packet.** | |
| Consent forms must be MS Word documents and follow the specific format outlined by the IRB. [**Click here**](https://irb.duhs.duke.edu/forms) to download a copy of the consent form template.  **Note:** Please do not edit the section of the footer that contains the Protocol ID, Continuing Review  and Reference Date fields. Those fields will be used to stamp the final consent form when it is approved by the IRB. If you want to add an internal version date, please put it in the header. |  |
| **Who will conduct the consent process with prospective participants?** | |
| Provide their role(s) in this study (PI, Study Coordinator, etc.):  Study Co-PIs and selected trained key personnel |  |
| **Who will provide consent or permission?** | |
| **(Select all that apply):**  Participant  Parent(s) or Legal Guardian(s)  Legally Authorized Representative (LAR) |  |
| **How much time will the prospective participant (or legally authorized representative) have between being approached about participating in the study and needing to decide whether or not to participate?** | |
| If you are not giving the person overnight to consider whether or not to participate, please justify. |  |

| The prospective participant or LAR will be given the opportunity to decide whether or not to participate, however long he/she/they deem necessary to make the decision. |  |
| --- | --- |
| **Where will the consent process occur?** | |
| In a private exam room in one of the iMIND study rooms. |  |
| **What steps will be taken in that location to protect the privacy of the prospective participant?** | |
| The prospective participant with the caregiver and/or LAR will be in the iMIND study room behind closed doors with the trained investigator/graduate student. |  |
| **How much time will be allocated for conducting the initial consent discussion, including presenting the information in the consent document and answering questions, with each prospective participant?** | |
| As long as necessary. |  |
| **What arrangements will be in place for answering participant questions before and after the consent is signed?** | |
| The participant will be given a copy of the consent which will include contact phone numbers if they have questions. They will also be given the study coordinator's phone number. |  |
| **Describe the steps taken to minimize the possibility of coercion or undue influence.** | |
| It will be explained to the prospective participant that the decision to participate in this study is completely their decision. No undue influence or coercion will be made by any of the study team members. |  |
| **What provisions will be in place to obtain consent from participants who do not read, are blind or who do not read/understand English?** | |
| Subjects who do not read or understand English will not be enrolled. For some cognitively impaired participants, a surrogate (LAR) for purposes of informed consent will be necessary, if the participants are deemed by the study team and/or their neurologist or treating physician via the Capacity Assessment to lack capacity to make an informed decision regarding informed consent and study participation. |  |
| **Do you plan to obtain written consent for the conduct of research?** | |
| Yes No |  |
| **Protected Health Information (PHI)** | |
| **Indicate how you intend to use potential subjects' Protected Health Information (PHI):** | |
| I will review, but not record, PHI prior to consent. I will record PHI prior to consent.  I do not intend to use PHI prior to consent.  I will record PHI without consent. (decedent research, database repository, chart review) |  |
| **Request for Waiver or Alteration of Consent and/or HIPAA Authorization** | |
| **Will the population include deceased individuals?** | |
| Yes No |  |

| **This waiver request applies to the following research activity or activities:** | |
| --- | --- |
| Scheduling of research activities in MaestroCare and/or the recording of PHI via telephone for screening purposes prior to obtaining written consent for the research.  Ascertainment (identification, selection) and/or recruitment of potential subjects while recording identifiable private information, such as protected health information (PHI), prior to obtaining the subject’s consent.  Conduct of the research project without obtaining verbal or written consent and authorization.  **Note: Answer the questions below as they pertain solely to PHI collected prior to consent.** |  |
| **Provide the following information:** | |
| **List the elements of informed consent and/or HIPAA authorization for which waiver or alteration is requested:**  Provide the rationale for each.  Patients may contact us after reading our IRB-approved Duke iMIND study flyers to learn more and see if they meet inclusion criteria. Waiver to ask them about their name, age, sex for scheduling purposes. And waiver to ask if they have diabetes or any eye conditions including prior retinal surgery to determine if they broadly meet inclusion criteria. Also requesting waiver to review clinic schedules of physicians and other providers at the Duke Memory Disorders Clinics and Duke neurology clinics at Duke Morreene Road in Durham, NC to review which patients might potentially be eligible for iMIND study participation. This would include reviewing their medical history.  We are requesting waiver for all elements of informed consent and HIPAA authorization.  **List the specific protected health information (PHI) to be collected and its source(s):**  (Note: PHI = health information + identifiers)  Name, age, sex, question about whether they have diabetes or any serious eye problems including prior retinal surgery to see if they meet basic inclusion criteria. |  |
| **Criteria for Waiver: The DUHS IRB may waive the requirement for informed consent and authorization if all of the following criteria are met:** | |
| Please respond to each item in the space below using protocol-specific language to provide justification:   a) **The research or clinical investigation involves no more than minimal risk to subjects:**   The information shared by the patients will be shared with the IRB-approved study team only on an as-needed basis to conduct the study – does not involve more than minimal risk.   b) **The waiver or alteration will not adversely affect the rights and welfare of the**   **subjects. Include a description of any measures to be taken to ensure that the rights and welfare of subjects will be protected:**  Participants (or LAR based on Capacity Assessment) will still sign the informed consent form once they come in to the study site.   c) **Whenever appropriate, the subjects will be provided with additional pertinent information after participation:**   All questions about the iMIND study will be answered.   d) **If this research activity relates to research involving deception, explain how subjects will be provided with additional pertinent information after study participation and what information will be provided. Otherwise indicate “not applicable”:** |  |

| Not applicable   e) **The use or disclosure of protected health information involves no more than minimal risk to the privacy of individuals, based on, at least, the presence of the following elements (e1. and e2.)**   **Demonstrate that the use or disclosure of PHI involves no more than minimal risk to the privacy of subjects by describing the plans requested below:**  **e1) An adequate plan to protect the identifiers from improper use and disclosure.**  **Describe the plan (how protection will be accomplished) and indicate where the PHI will be stored and who will have access:**  The clinic schedules for the physicians and other providers at Morreene Road will be reviewed by Dr.  Fekrat or Dr. Grewal or trained physician study staff through their  secure Epic login to determine which patients might be potentially eligible for iMIND study  participation. Duke DEDUCE search query will be conducted & used for recruitment as well along with Duke PACE. The clinic physicians and providers will be notified of any of their own clinic patients who may be potentially eligible for the study via send secure Duke email so that they can potentially mention the iMIND study to them during their clinic visit to gauge whether they are interested in learning more about the iMIND study by one of the iMIND study staff on that same day. PHI will not be stored.  For controls, once patients contact us via password-protected Duke iMIND email, we will contact them by email or phone to share information about the iMIND study using IRB-approved language to gauge further interest. PHI will not be stored as part of the phone call or email.  **e2) An adequate plan to destroy the identifiers at the earliest opportunity consistent with conduct of the research, unless there is a health or research justification for retaining the identifiers or such retention is otherwise required by law.**  **Describe the plan (how and when identifiers will be destroyed and by whom). If there is a health or research justification for retaining the identifiers or such retention is otherwise required by law, provide the reason to retain identifiers:**  Patients who come into the iMIND study site will be consented and their study-related information recorded as per study protocol. No other information will be stored.  **e3) Adequate written assurances that the protected health information will not be reused or disclosed to any other person or entity except (i) as required by law, (ii) for authorized oversight of the research study, or (iii) for other research for which the use or disclosure of PHI would be permitted by the HIPAA Privacy Rule. By electronically signing this submission, the PI provides this written assurance:**  The PI attest to the above by applying his electronic signature.   f) **The research could not practicably be conducted or carried out without the waiver or alteration:**   Explain why informed consent/authorization can not be obtained from subjects.  Individuals who contact us after seeing an iMIND study flyer or learning about our study from other sources may not yet be enrolled but have questions about the study and want to learn more and whether or not they are eligible for participation.   g) **The research could not practicably be conducted or carried out without access to and use of the protected health information:**   Individuals who contact us after seeing an iMIND study flyer or learning about our study from other sources may not yet be enrolled but have questions about the study and want to learn more and whether or not they are eligible for participation.   h) **For research using biospecimens or identifiable information, the research could not practicably be carried out without access to and use of the protected health information:**   Individuals who contact us after seeing an iMIND study flyer or learning about our study from other sources may not yet be enrolled but have questions about the study and want to learn more and whether or not they are eligible for participation. |  |
| --- | --- |
| **Waiver of Documentation of Consent and HIPAA Authorization for Scheduling in MaestroCare and/or the** | |

| **recording of PHI via telephone for screening purposes:** | |
| --- | --- |
| **These research activities prior to obtaining written consent for the study presents no more than minimal risk of harm to subjects:**  True False  **These are procedures for which written consent is normally not required outside of the research context:**  True False  **An IRB-approved phone script will be used to obtain verbal consent from subjects for scheduling and/or screening prior to obtaining written consent for the study:**  True False |  |
| **IRB Notification of Decedent Research** | |
|  | |
| In accordance with 45CFR164.512(i)(1)(iii), the IRB must approve research involving decedent's private health information. In order for the IRB to make the determination, please respond to each item in the allotted space below, using protocol-specific language to provide justification. |  |
| **Provide a brief, meaningful description of the protected health information for which use or access has been determined to be necessary:** | |
| Name, age, sex, medical and ophthalmic history, and images that were already captured during participation in Pro00082598. |  |
| **Check each statement below to attest to your knowledge that:** | |
| The PHI to be used will be used solely for research. All subjects to whom this form applies will be dead. The PHI is necessary for the research.  You will not disclose such PHI (share with anyone from outside DUHS or the SOM/SON) without first removing all direct identifiers, and also all indirect identifiers including information about the patient's age if greater than 89 years (state instead that the age is 90+ years), any dates of health-related events, and any patient's address more specific than state or 3 digit zip code (thus the data become de-identified).  Alternatively, you may choose to disclose this PHI, but, if so, you declare that you will maintain an accounting of this disclosure (commonly referred to as "tracking" the disclosure) for the patient's next of kin if requested. |  |
| **Devices** | |
| **Include all devices being evaluated in this study:** | |
| Include all devices being evaluated in this study to determine their safety or effectiveness, and include information about a humanitarian use device where requested. Also add devices without an IDE here, including any Humanitarian Use Device that does not require an IDE because it is to be used according to its FDA approved product labeling, and its safety or effectiveness is not being evaluated. |  |

|  |  |  | **Will the device to be evaluated or** | **IDE** |
| --- | --- | --- | --- | --- |

| **Who will be responsible for the storage, inventory and control of the device to be evaluated or the Humanitarian Use Device?** | |
| --- | --- |
|  |  |

| **View Details** | **Device Name** | | | **Is the Device FDA Approved** | | **the Humanitarian Use Device be manufactured at Duke?** | **/Compassionate Use Request Number** |
| --- | --- | --- | --- | --- | --- | --- | --- |
|  | Optos California Camera | | | Yes | | No |  |
| Device Source | | OPTOS, INC | | | | | |
| CMS Category | |  | A |  | B |  |  |
| Is the device provided to subject free of charge? | | Yes | | | | | |
| Is this a HUD (HDE)? | | No | | | | | |
| HDE Number | |  | | | | | |
| Is the Device FDA Approved | | Yes | | | | | |
| Will the device to be evaluated or the Humanitarian Use Device be manufactured at Duke? | | No | | | | | |
| Do you have an IDE number for this device? | | No | | | | | |
| IDE/Compassionate Use Request Number | |  | | | | | |
| IDE Holder | | N/A | | | | | |
| IDE Details | |  | | | | | |
| In the opinion of the sponsor, select the level of risk associated with this device | | No Significant Risk | | | | | |
|  | Zeiss Cirrus HD-5000 with AngioPlex | |  | Yes |  | No |  |
| Device Source | | Carl Zeiss Meditec, Inc. | | | | | |
| CMS Category | |  | A |  | B |  |  |
| Is the device provided to subject free of charge? | | Yes | | | | | |
| Is this a HUD (HDE)? | | No | | | | | |
| HDE Number | |  | | | | | |
| Is the Device FDA Approved | | Yes | | | | | |
| Will the device to be evaluated or the Humanitarian Use Device be manufactured at Duke? | | No | | | | | |
| Do you have an IDE number for this device? | | No | | | | | |
| IDE/Compassionate Use Request Number | |  | | | | | |
| IDE Holder | | N/A | | | | | |
| IDE Details | |  | | | | | |
| In the opinion of the sponsor, select the level of risk associated with this device | | No Significant Risk | | | | | |

| The Co-PIs and the study team members.  The devices are being used according to FDA-approved product labeling and thus its safety and effectiveness are not being evaluated. |  |
| --- | --- |
| **Where will the device to be evaluated or the Humanitarian Use Device be stored?** | |
| Duke Neurological Disorders Clinic at 932 Morreene Road, Rm 216, Durham, North Carolina, 27705, USA. |  |
| **Who will be responsible for giving or administering the device to be evaluated or the Humanitarian Use Device to the research subject?** | |
| One or more members of the iMIND study team. |  |
| **From where will the device to be evaluated or the Humanitarian Use Device be dispensed?** | |
| Carl Zeiss Meditec and Optos, Inc. |  |
| **At the completion of this research study, what will be done with the unused or returned device or the Humanitarian Use Device?** | |
| The Carl Zeiss HD-5000 with AngioPlex device is owned by Duke. The Optos California camera which is a loaner (loaner agreement has been completed) will be wiped clean of any and all Duke patient data and returned to Optos. |  |
| **Privacy and Confidentiality** | |
| **Explain how you will ensure that the subject's privacy will be protected:** | |
| Consider privacy interests regarding time and place where subjects provide information, the nature of the information they provide, and the type of experience they will be asked to participate in during the research.  Subjects are recruited from Duke Health clinics including the memory and neurology clinics and from the Duke Alzheimer’s Disease Research Center (ADRC), Duke Aging Center, as well as from DEDUCE searches and IRB-approved flyers shared with Duke Dementia Family  Support, NC Registry for Brain Health, and community sources. Subjects are contacted by telephone using IRB-approved phone script or by Duke iMIND email via send secure using IRB-approved email verbiage.  The Duke guidance on emailing potential study participants (*Best Practices for Email Use for Clinical Research*) will be followed by all study staff. Interested subjects will be brought to the iMIND  study room where they will meet with the study investigator/graduate medical student/trained study staff in private, with their LAR as indicated.  The investigator/graduate student/staff will explain the study and answer any questions the patient or LAR might have. If the patient agrees to participate, the study investigator  /graduate student will consent the patient or LAR (based on Capacity Assessment) and  continue with all necessary paperwork for enrollment at that time. After the study visit is complete, the participant, and LAR as indicated, will depart.  Each subject will be assigned a unique study code that will allow identification for study participation. Stored study data and images will be de-identified.  The patients’ identities will not be identifiable in any publications or presentations resulting from this investigation.  Although we have taken steps to secure protected health information (PHI) and protect the patients' privacy, there are theoretical risks to the patient’s confidentiality.  Images shared with collaborating institutions for the purposes of image analyses not available at Duke will be deidentified and shared through password-protected DukeBox with data sharing/transfer agreements in place. The purpose of image sharing is for analyses of parameters within images that Duke does not have the expertise to complete.  De-identified images and associated quantitative data will be analyzed in Duke Compute Cluster (DCC). |  |

| **Describe how research data will be stored and secured to ensure confidentiality:** | |
| --- | --- |
| How will the research records and data be protected against inappropriate use or disclosure, or malicious or accidental loss or destruction? Records and data include, for example, informed consent documents, case report forms or study flow sheets, survey instruments, database or spreadsheets, screening logs or telephone eligibility sheets, web based information gathering tools, audio/video/photo recordings of subjects, labeled specimens, data about subjects, and subject identifiers such as social security number.  Subjects will be promptly de-identified via the use of a separate document stored on DukeBox correlating subjects’ name and Duke medical record numbers with a study ID assigned for the sole purposes of this study. At the end of data collection, that key will be destroyed. Files will not be shared with non-study personnel.  Data will be stored on a secure server in password-protected REDCap. Images will be stored in the password-protected imaging machines from which they were acquired, password-protected DukeBox, and password-protected computers in the Duke iMIND study rooms.  De-identified UWF fundus photographs with all elements of identifiable data removed will be shared via password-protected DukeBox with University of Edinburgh for analysis of retinal vessel parameters using VAMPIRE software [**(http://vampire.computing.dundee.ac.uk/publications.html).**](http://vampire.computing.dundee.ac.uk/publications.html))  De-identified OCT, OCTA, and UWF images with all elements of identifiable data removed will also be used in the DCC for training, validation, and testing of machine learning algorithms for detecting these conditions.  De-identified OCT images with all elements of identifiable data removed will be shared via password-protected DukeBox with Tan Tock Seng Hospital in Singapore password-protected COIN software platform for the measurement and analysis of choroidal structure and vascularity.  We may need to share the deidentified images with the US Food and Drug Administration (FDA) for approv al of a diagnostic index or machine learning  model to diagnose AD or any of these neurodegenerations. These algorithms are for exploratory purposes only and will not be used to assess or treat subjects in this study.  For the purpose of review prior to publication, the following institutions may also review the de-identified images:  TAN TOCK SENG HOSPITAL PTE LTD - Prof Rupesh Agrawal Queen's University of Belfast - Imre Lengyel, M.D. University of Edinburgh, Scotland – Tom J MacGillivray PhD |  |
| **Application Questions Complete** | |
| **Please click Save & Continue to proceed to the Initial Submission Packet.** | |
| The Initial Submission Packet is a short form filled out after the protocol application has been completed. This is an area to attach protocol-related documents, consent forms, and review the application. |  |
